# Greener reactants, renewable energies and environmental impact mitigation strategies in pyrometallurgical processes: A review

**DOI:** 10.1557/s43581-022-00042-y

**Published:** 2022-09-07

**Authors:** Jean-Philippe Harvey, William Courchesne, Minh Duc Vo, Kentaro Oishi, Christian Robelin, Ugo Mahue, Philippe Leclerc, Alexandre Al-Haiek

**Affiliations:** 1 Department of Chemical Engineering, Centre for Research in Computational Thermochemistry (CRCT), Polytechnique Montréal, Station Downtown, Box 6079, Montreal, QC H3C 3A7 Canada; 2 R & D and engineering services, LAh Services G.P., Montreal, QC H4N 0A7 Canada

**Keywords:** Metal, materials sourcing, chemical reaction, environmental impact, sustainability, lifecycle

## Abstract

**Abstract:**

Metals and alloys are among the most technologically important materials for our industrialized societies. They are the most common structural materials used in cars, airplanes and buildings, and constitute the technological core of most electronic devices. They allow the transportation of energy over great distances and are exploited in critical parts of renewable energy technologies. Even though primary metal production industries are mature and operate optimized pyrometallurgical processes, they extensively rely on cheap and abundant carbonaceous reactants (fossil fuels, coke), require high power heating units (which are also typically powered by fossil fuels) to calcine, roast, smelt and refine, and they generate many output streams with high residual energy content. Many unit operations also generate hazardous gaseous species on top of large CO_2_ emissions which require gas-scrubbing and capture strategies for the future. Therefore, there are still many opportunities to lower the environmental footprint of key pyrometallurgical operations. This paper explores the possibility to use greener reactants such as bio-fuels, bio-char, hydrogen and ammonia in different pyrometallurgical units. It also identifies all recycled streams that are available (such as steel and aluminum scraps, electronic waste and Li-ion batteries) as well as the technological challenges associated with their integration in primary metal processes. A complete discussion about the alternatives to carbon-based reduction is constructed around the use of hydrogen, metallo-reduction as well as inert anode electrometallurgy. The review work is completed with an overview of the different approaches to use renewable energies and valorize residual heat in pyrometallurgical units. Finally, strategies to mitigate environmental impacts of pyrometallurgical operations such as CO_2_ capture utilization and storage as well as gas scrubbing technologies are detailed. This original review paper brings together for the first time all potential strategies and efforts that could be deployed in the future to decrease the environmental footprint of the pyrometallurgical industry. It is primarily intended to favour collaborative work and establish synergies between academia, the pyrometallurgical industry, decision-makers and equipment providers.

**Graphical abstract:**

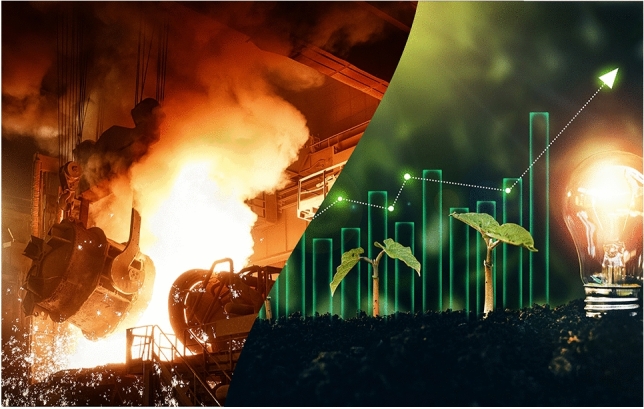

**Highlights:**

A more sustainable production of metals using greener reactants, green electricity or carbon capture is possible and sometimes already underway. More investments and pressure are required to hasten change.

**Discussion:**

- Is there enough pressure on the aluminum and steel industries to meet the set climate targets?
- The greenhouse gas emissions of existing facilities can often be partly mitigated by retrofitting them with green technologies, should we close plants prematurely to build new plants using greener technologies?
- Since green or renewable resources presently have limited availability, in which sector should we use them to maximize their benefits?

## Introduction

The fast modernization of our society coupled with its linear growth since the 1960s puts tremendous pressure on virtually all the major industrial sectors linked to energy, natural resources, transportation and goods. As a direct consequence, the extraction of natural resources and their processing at large scale has been running at an infernal pace for decades. Iron (1.51 Bt/year), aluminum (63.23 Mt/year), manganese (20.18 Mt/year), chromium (15.29 Mt/year), copper (20.47 Mt/year), zinc (12.64 Mt/year), titanium (7.35 Mt/year), lead (4.64 Mt/year) and nickel (2.26 Mt/year)^1^ are among the commodity metals needed to produce not only buildings and bridges but also smart-phones and electric cars. This intense metal production coupled to our societal cravings for electricity, heat generation and transportation comes with important environmental impacts. In 2021, an estimated 36.4 billion metric tons of CO_2_^2^ emissions were released to the atmosphere. The use of energy in heavy industry accounts for 24.2% of these emissions while transport is responsible for 16.2%.^3^ Mining operations, which are at the heart of the primary metal production, released alone about 8% of these CO_2_ emissions^4^ (with a large portion associated with comminution operations^5^) and so did the pyrometallurgical production of metals (ferrous and non-ferrous). A significant portion of these emissions related to pyrometallurgical processes are coming from the iron and steel industry while only a minor fraction is caused by other non-ferrous metal production (notably aluminum with about 1% of the total emissions.^6^) By considering an average CO_2_ footprint of 1.8 kg per kg of steel produced,^7^ the steel industry is responsible for more than 7% of the global worldwide anthropogenic emissions. This estimation should be taken with care as the quantification of the environmental impacts of a given metal obtained from life cycle analysis (LCA) is influenced by many parameters such as the functional unit under study, the system boundaries, the unit operations considered in the process (and their operating parameters) and the location of the process (available energy mix). The main CO_2_ emissions attributed to the pyrometallurgical industry were related to (1) the use of carbon materials for reduction (pyro-processes) and heating (furnaces), (2) electric power production (heating and electrometallurgy), (3) transportation and (4) carbonate decomposition (ciment and glass industries are other significant contributors), among others.

Traditionally, the primary production of metals involved the extraction of a valuable element from minerals (in the form of oxides, sulfides, sulfates, carbonates and hydroxides) locked into a rock that also contain gangue materials (usually silicates). Because of that, the economic profitability of these processes is directly related to the rate at which these ores or concentrates are processed. Typical smelting technologies can produce from 1 tonne per day (ex.: a Hall–Heroult electrolysis cell operating at 500 kA can produce about 4000 kg of aluminum per day^8^) up to thousands of tonnes per day (ex.: blast furnaces can produce about 15,000 tonnes of iron per day^9^). Cheap and abundant reactants such as coal and air have been at the heart of pyrometallurgical processes for centuries. They are used to calcine, roast, smelt, convert and refine metals. The strong affinity of carbon and sulfur for oxygen is of prime technological importance as it can (1) reduce oxides to produce metals, (2) roast sulfides to produce oxides, sulfates or metals or (3) be burnt in the presence of O_2_ to release energy in the form of heat or work. These traditional reactants are involved in chemical reactions that release CO_2_, NO_x_ and SO_2_. Environmental concerns related to acid rains in the 70’s led to SO_2_ industrial capture systems (which produce sulfuric acid H_2_SO_4_) and improved combustion technologies that use oxygen-enriched air and catalysts.

More recently, the accumulation of end-of-life technological products (which contain metals and alloys, semi-conductors, oxides and polymers) that contributed to our industrial development (such as cars, appliances, computers, cell phones and batteries) paved the way to the elaboration of recycling strategies that involve primary metal processes: electronic appliances can be recycled in copper or lead smelting operations; steel can be processed in basic oxygen furnaces to modulate their heat balance; metal hydride batteries can be recycled in nickel smelting operations. Re-melting furnace technologies also emerged from the availability of well-sorted aluminum, iron and copper scrap. These re-melting furnaces have been fueled by oil, diesel and more recently by natural gas. They also contribute to the footprint of the pyrometallurgical industry by emitting CO_2_, NO_x_ and SO_2_.

Humanity faces tremendous challenges if it wants to sustain its metal production and recycling. Even though decarbonization is the main direction to lower the impact of pyrometallurgical processes, its application comes with a plethora of challenges: (1) carbon materials are the only natural reducing agents available on earth, (2) the combustion of carbon-based materials releases almost instantaneously the energy required to maintain the high temperature of pyrometallurgical processes, (3) solid carbon materials are chemically inert to many aggressive high-temperature environments, (4) solid carbon materials are good electric and heat conductors. Bio-sourced carbon materials obtained from the treatment of industrial waste (such as the pulp and paper as well as the wood construction industries) may provide an alternative source of carbon materials with a smaller environmental footprint (i.e. shorter carbon cycle). A further decarbonization step can be taken by using hydrogen for reduction as well as heating operations.

In pyrometallurgical processes, high power input is required when it comes to drying, calcining, melting and even reducing tonnes of materials per day. When available, green power grids can offer the following alternative technologies to conventional oil/gas burners to achieve these heat-related operations: plasma torch, resistance heating, induction and micro-waves are some of the emerging technologies offered to the industry. Green electric power also draws a lot of attention when it comes to the clean production of hydrogen to fuel cars, trucks and furnaces. It is worth mentioning that hydrogen is not the only green fuel which can be produced from electrochemical methods. Ammonia, as well as aluminum and other reactive metals such as sodium and lithium can also be produced from electric-based processes. In recent years, even iron has been targeted as a potential fuel^10^ which could also be produced from direct molten oxide electrolysis. These metals all have a high specific internal energy stored in their metallic bonds. In fact, the amount of stored energy in these materials competes with the one of pressurized hydrogen. These non-conventional metallic fuels may one day power not only spacecrafts but also thermal power plants.^10,11^

This paper first describes the current state of the various pyrometallurgical operations which are commonly used to produce industrial metals such as iron, aluminum, nickel and copper. These involve drying and calcining, roasting, reduction and smelting as well as refining unit operations. The environmental impacts of the primary production of these metals obtained from LCA analyzes found in the literature are then presented and quantified to identify the critical unit operations in terms of CO_2_ emissions and energy consumption. We then explore the recent advances related to the use of greener reactants such as bio-fuels, bio-char, hydrogen, ammonia and various scrap streams that are being integrated in pyrometallurgical processes to lower their environmental footprint. This paper also reviews the use of renewable energies in high-power operations such as furnaces and high temperature electrolysis processes, the valorization of residual energy available in various output streams (such as off-gases and slags) as well as the mitigation strategies linked to CO_2_ capture and utilization for processes that cannot avoid the use of carbonaceous materials. Computational thermochemistry is also used throughout the work (via the use of the FactSage package) to support the discussion. This original review paper brings together for the first time all potential strategies and efforts that could be deployed in the future to decrease the environmental footprint of the pyrometallurgical industry. It is primarily intended to favour collaborative work and establish synergies between academia, the pyrometallurgical industry, decision-makers and equipment providers. Finally, it is to be mentioned that a special focus is put throughout the work on the primary and secondary production of iron and aluminum as they are the main CO_2_ emitters in the pyrometallurgical industry.

## Pyrometallurgical operations: Current state

Most of the actual pyrometallurgical processes used in the industry are based on relatively mature technologies: the injection of natural gas and oil via the tuyeres of blast furnaces used for the production of pig iron has been introduced in the 1960s^12^; prebaked carbon anodes used in Hall–Héroult cells for the primary production of aluminum have been developed since the 1920s^13^ and the environmental efficiency of this process was discussed.^14^ The Noranda process implemented in Quebec and originally designed for the primary production of copper from a copper sulfide concentrate has also been processing electronic waste material since the 1980s^15^; electric arc furnaces used in steelmaking to melt DRI pellets and iron scrap with a specific power that can reach power of 1000 kVA/ton were already available in the 1980s.^16^

**Table 1:** Common reduction and smelting processes of industrial metals.

Metal	Process	Reactants	Reducing agent	Extra energy
Fe	BF	Fe_2_O_3_/flux, O_2_/air	Coke, coal	Coke, coal
	DRI	Fe_2_O_3_/flux	H_2_, CH_4_, CO	
	EAF	Scrap, DRI	Coke, coal	Coke, coal, electricity
Fe–Si	EAF/BF	Silica, scrap iron	Coke, coal, anode	Coke, coal, electricity
Fe–Mn	EAF/BF	MnO_2_, Fe_2_O_3_	Coke, coal, anode	Coke, coal, electricity
Fe–Cr	EAF	FeCr_2_O_4_	Coke, coal, anode	Coke, coal, electricity
Fe–Ni	RKEF	Nickel laterite	Coke, coal, anode	Coke, coal, electricity
NPI	BF	Nickel Laterite	Coke, coal	Coke, coal
Al	HH	Alumina, cryolite	Electricity, anode	Electricity
Mg	Pidgeon	Dolomite, CaO, MgO	Si, Fe-Si	Coal
	Modified Pidgeon	Dolomite, CaO, MgO	Si, Fe-Si	Electricity
	MTMP	Calcined dolomite	Al, Fe-Si	Electricity
	Electrolysis	MgCl_2_, KMgCl_3_	Electricity	Electricity
Ni	Roast-RS	Sulfide ores, silica, O_2_/air		fossil fuel
Ni	FS	Sulfide ores, silica, O_2_/air		fossil fuel
Cu	Bath smelting	Sulfide ores, silica, O_2_/air	methane (refining)	fossil fuel
	TSL	Sulfide ores, silica, O_2_/air		fossil fuel
	FS	Sulfide ores, silica, O_2_/air		fossil fuel

*DRI* direct reduction iron, *BF* blast furnace, *BOF* basic oxygen furnace, *EAF* electric arc furnace, *NPI* Nickel pig iron, *HH* Hall–Héroult, *MTMPM* Mintek thermal magnesium process, *FS* flash smelting, *RKEF* rotary Kiln-electric arc furnace, *RS* reductive smelting, *TSL* top submerged lance.

### Drying and calcining furnaces

The ores which contain valuable minerals are typically processed to concentrate the metal of interest and to modulate the size of the solid particles to be injected in subsequent pyrometallurgical units. These mineral processing operations, which often use water, lead to wet concentrates that need to be dried.^17^ The behavior of fine particles directly injected in furnaces (such as flash smelters) is highly impacted by the presence of water. Drying is performed using one of the following equipments:

- *Rotary drums*-These kilns have been extensively used in high-productivity processes for the calcination of : (1) carbonates in the cement production; (2) hydrated alumina obtained from the Bayer process (to obtain dry alumina that feeds Hall–Héroult cells)^18^; (3) nickel laterite concentrates which then feed electric arc furnaces^19^; (4) petroleum coke^20^ which involves drying, devolatilization and densification steps. In the case of lithium extraction from spodumene, calcination is performed in a direct-fired rotary furnace.^21^ The calcination step involves a significant volume change called decrepitation. In conventional kilns, hydrocarbon-based burners are used to provide the energy to dry and calcine the feed material. Both direct and indirect heating furnaces exist, the latter being more exploited in recent years due to the improvement of alloys and refractory resistance to high temperature. Such indirect heating systems also permit the control of the atmosphere of the furnace. It is to be noted that the rotation of the reactor induces erosion of the steel surface of the vessel (or refractory lining if the temperature is too high), which leads to frequent maintenance.
- *Steam dryers*-This technology introduced in 1990 is used to dry copper, nickel, lead and zinc concentrates as well as other abrasive materials such as lignite. Hot steam is passed in sealed tubes instead of combustion gases. This steam is condensed as it transfers its energy to the charge outside the tubes. One important advantage of steam dryers over conventional direct-heating gas-burner rotary furnaces is the possibility to control the inertness of the atmosphere of the reactor to prevent oxidation.^22^ In the Ni and Cu industries, waste heat boilers^23^ and acid plants^24^ are generating steam to be used in these steam dryers to directly valorize residual energy.
- *Fluidized bed and flash dryers*-More recently, many scientists and engineers started to explore the possibility to calcine fine concentrates via fluidized bed reactors (which are more compact technologies^25^) and flash furnaces.

### Roasting furnaces

Roasting is a pyrometallurgical operation changing the chemistry and phase assemblage of a feed material. It usually involves the injection of reactants such as air, chlorine and carbon-based materials in conjunction with the use of burners. Roasting can be associated with either exothermic or endothermic reactions. Actual roasting operations in pyrometallurgy include: (1) iron ore induration^26^; (2) Zn sulfide conversion to ZnO using O_2_-enriched air blowing^27^; (3) carbo-chlorination of titanium concentrate that transforms the titanium oxide into titanium chloride.^28^ As for calcining furnaces, roasters used in pyrometallurgical processes are conventionally heated via burners and have a rotary drum system.

### Reduction and smelting reactors

At the heart of pyrometallurgical operations to ultimately extract metals are the reduction reactors and smelters. Reduction reactors such as Direct Reduction Iron (DRI) furnaces are operated using feed materials that stay in the solid state. These low-temperature operating conditions drastically lower the energy requirements of the furnace and wear of its surface in contact with the charge. Carbon-based materials (such as natural gas^29^ and coal^30^) as well as hydrogen^29^ are used in these reactors to remove oxygen in the form of CO, CO_2_ and H_2_O gaseous species. The generation of liquid phases such as slags, molten matte and metallic melts is promoted in smelting technologies. Smelters use both reducing (such as coke in blast furnaces) and oxidizing reactants (such as oxygen-enriched air used in tuyere smelting of copper sulfides^31^) to produce the targeted metal. Moreover, electrons can also be used as a reducing agent in electrochemical smelters (a field called electro-metallurgy). The presence of liquid phases in smelters induces many advantages over solid-state reactors: (1) it increases the kinetics and diffusion mechanisms; (2) it facilitates the physical separation of the valuable metallic melt from the impurities which are typically partitioned and collected in a slag phase; (3) it improves the heat transfer inside the reactor and allows a more homogeneous temperature profile. Challenges associated with the presence of high temperature liquids are the corrosion of the materials used to contain the feed as well as the potential volatilization and atmospheric release of toxic species (such as Pb and As), and the production of environmentally non-friendly gaseous species such as NO_x_. Table 1 summarizes some of the current reducing and smelting technologies that are used in the primary production of industrial metals.

#### Electric arc furnaces

Iron and ferroalloys (e.g., Fe–Mn, Fe–Cr, Fe–Si)^32^ are the most produced technological metals and alloys on earth. They are the basic ingredients for the production of steel which finds applications everywhere. The production of these ferroalloys often involves the use of an electric arc furnace. This type of reactor powered by electricity heats the charge via the generation of electric arcs/plasma (in non-submerged electrodes configuration) as well as via the Joule effect in the submerged electrode configuration (when the charge to melt has a high electrical resistance). Fossil fuel burners are also used to complement heating in colder zones. The graphite electrodes, which can be prebaked or baked *in situ* (i.e. Soderberg electrodes), are consumed during the furnace operation to produce gaseous CO. Coke as well as another cheap carbon source (coal) can also be injected in the molten slag to further reduce it and lower graphite electrode consumption. Both direct-current and alternative-current technologies exist. Here is a list of metals and ferroalloys produced via EAF:^33^

- *Fe–Cr*: Chromite smelting (FeO$\cdot$Cr_2_O_3_)
- *Fe–Si*: Silica smelting (iron, calamine (iron oxide scrap), SiO_2_)
- *Fe–Ni*: production from laterites
- *Metal recovery from slag*: Cu, Co
- *Platinum group metals*

#### High-temperature electrolysis cells

The extraction of highly reactive metals such as Al, Mg, Na, and Li is a challenging task. The high thermodynamic stability of their respective oxides requires using extreme temperature conditions for their carbo-reduction. Moreover, the formation of highly stable carbides (such as Al_4_C_3_^34^ in the case of alumina carboreduction) and volatile species (such as Al_2_O and AlO) are other problems that may prevent the use of such reduction processes. One alternative to circumvent these problems is to use high-temperature electrolysis. Here, electrons are used to reduce the metallic cations available in a molten electrolyte. A conventional electrolysis cell is made of an anode and a cathode which are both submerged in a liquid electrolyte. By flowing an external electric current in the electrochemical system for some imposed potential difference between the two separated electrodes, metal (in the form of a liquid or a solid) can be ultimately produced at the cathode. At the anode, an oxidation reaction releasing free electrons is promoted. A key feature of an electrolysis cell design is the selection of the optimal molten electrolyte that ensures the ionic transport within the cell. Electrolytes actually used for high-temperature electrolysis include both molten chlorides^35^ and molten fluorides. Ideally, the selected electrolyte directly dissolves the concentrate in the form of anionic complexes and metal cations. This is the case of some molten fluorides such as cryolite (Na_3_AlF_6_) used in the Hall–Héroult process which significantly dissolves alumina.^36^ In comparison, the solubility of MgO in molten fluorides is rather small^37^ and prevents the use of such an electrolyte for magnesium production. Even though slags appear as logical molten electrolytes to dissolve oxide concentrates, their non-negligible electrical conductivity along with their high liquidus temperatures and corrosiveness prevent their industrial use in electrolysis cells at this time. Molten chlorides which are abundant and cheap are interesting electrolytes.

### Refining technologies

A tight control of the presence of impurities and of the overall chemistry of the metal/alloy is required when one wishes to tune their performance in service. As examples, (1) copper used in electrical applications needs to be exempt of impurities and requires at least 99.9 % (wt) of purity, which is obtained from the low-temperature electro-refining of copper anodes^38^; (2) steel toughness is greatly impacted by the presence of non-metallic inclusions^39^ whose presence can be reduced by the deoxidation of the molten metals using aluminum powder and other deoxidizers; (3) the presence of hydrogen in molten aluminum is critical as its virtually zero solubility in the solid-state leads to the formation of porosities upon casting^40,41^ which can be avoided by degassing the liquid aluminum via bubble methods or vacuum heating.^42^ As can be seen from this list of examples, there is an abundance of technologies for each industrial metal that cannot be exhaustively reported here. It should be highlighted that the refining units typically take advantage of the already hot and molten metal to be refined, thus lowering the energy requirements of the process. In the steel industry, the temperature of the melt to be refined can be maintained using electrically powered ladle furnaces, which require substantially less energy than EAF used to melt the charge.^43^ Similar ladle furnaces are also used to refine molten ferronickel, which typically contains sulfur and phosphorous that need to be removed.^44^ Other molten iron refining and alloying units exploit induction heating to promote electromagnetic stirring. Induction stirring is also used in continuous casting operations.^45^ Apart from the use of energy to maintain the temperature of the reactor, refining units also consume/use chemicals such as argon (to remove volatile impurities such as hydrogen from molten iron^46^), fluxes to promote the formation of a slag^47^ as well as oxygen (to oxidize reactive impurities and transfer them to a slag phase).

**Table 2:** Summary of the LCA analyzes available in the literature on pyrometallurgical processes.

Metal	Country	Route	Global warming potential; Energy demand (kg CO_2_ eq/FU;kWh/FU)						Refs.
Fe			Mining	Sintering	BF	BOF	Coke oven	Total	
	Australia	BF-BOF	n/a					2.3;6.39	^48^
	Poland	BF-BOF	n/a					2.46;9.84	^49^
	Italy	BF-BOF	n/a	0.327;0.53	0.663;3.72	0.242;0.22	0.357;1.03	1.59;5.5	^50^
	China	BF-BOF	n/a	378;–	1.29;–	0.221;–	0.185;–	2.04;5.45	^51^
	Poland	EAF						0.91;8.07	^49^
	China	EAF						0.86;8.25	^51^
	China	DRI-EAF						1.23;19.87	^52^
	–	DRI-EAF						1.5;22	^53^
Al			Mining	Alumina	Anode	HH	Casting	Total	
	Australia	HH						22.4;58.6	^48^
	Global	HH	< 0.1;–	3.8;–	0.6;–	11.9;–	0.2;–	16.5;52.8	^54^
	ROW	HH	< 0.1;–	2.8;–	0.6;–	7.2;–	0.2;–	10.8;42.5	^54^
	USA	HH	0.079;0.296	1.23;4.93		10.91;28.6	0.27;1.13	12.5;35	^55^
	China	HH	0.115;0.344	3.51;12.2	0.439;3.78	17.3;55.3	0.475;1.63	21.8;73	^56^
	China	HH	0.028;0.120	2.88;10.6	0.333;3.5	1.66;14.8	0.00748;0.417	4.91;29.4	^56^
	China	HH	1.073;–	2.7115;–	1.2035;–	9.1785;–	0.3335;–	14.5;–	^57^
	China	Recycling						0.72;–	^58^
	China	Recycling		0.215;–	0.215;–	0.50;–		0.93;–	^57^
	China	Recycling		0.060;0.23	0.114;0.44	0.382;1.49	0.101;0.39	0.658;2.56	^59^
FeNi			Mining	Extraction	Smelting	Refining	Other	Total	
	Greece	RKEF	1.89;6.4^a^	0.76;2.8	7.08;22.4	2.88;11.1		12.6;42.8	^60^
	–	RKEF	–;0.97	–;4.52	–;27.32	In smelting	–;6.21	8.43;39.15	^61^
	–	RKEF						6;30.6	^62^
	–	RKEF	0.486;2.22	0.972;2.67	10.57;39.07	In smelting	0.122;0.443	12.15;44.28	^63^
Ni			Mining	Extraction	Smelting	Refining	Other	Total	
	Australia	FS-EW						11.4;31.7	^48^
	–	FS-EW	1.40;6.24	0.74;4.07	4.57;25.6	1.27;7.29	− 0.33;− 2.91^b^	7.64;40.8	^61^
	–	FS-EW						14;48.3	^62^
	–	FS-EW	1.69;6.56	1.95;8.52	7.15;36.7	1.69;12.5	0.52;1.31	13;65.6	^64^
Cu			Mining	Concentration	Smelting	Refining	Other	Total	
	China	BS-ER						1.91;8.82	^65^
	Australia	FS-ER						3.3;9.17	^48^
	Chile	FS-ER	0.9;2.24	2.2;3.06	1.9;2.50	0.6;0.717	0.4;0.506	6.0;9.02	^66^
	China	FS-ER						5.88;22.7	^67^
	Sweden	FS-ER						4.75;46.7	^68^
	China	Recycling						0.68;3.42	^65^
	China	Recycling						1.59;6.69	^67^

The functional unit (FU) is 1 kg of metal.

*ROW* rest of world (minus China), *BF-BOF* blast furnace-basic oxygen furnace, *EAF* electric arc furnace, *HH* Hall–Héroult, *RKEF* rotary Kiln-electric arc furnace, *FS* flash smelting, *EW* electrowinning, *BS* bath smelting, *ER* electrorefining.

^a^Includes ore beneficiation.

^b^Energy credit from sulfuric acid.

## LCA analysis in pyrometallurgy

Life cycle analysis (LCA) is one of the most important tools to quantify the impacts of our industrial processes on the environment. In fact, the change of paradigm in the primary production and recycling of metals will be directly connected to LCA. More specifically, impact displacement is a major element to quantify when it comes to emerging technology integration. For this review, the impact of processes on climate change from greenhouse gas (GHG) emissions is at the forefront along with energy and electricity demand. As the focus is put on the metallurgical side of the production process, cradle-to-gate (i.e. from extraction to arrival at the end-user, but excluding impacts related to the use and end-of-life) LCA is pertinent for the primary production of metals with the boundaries encompassing ore mining and preparation, smelting, and refining. The common functional unit (FU) for such studies is 1 kg or 1 metric ton of metal. A list of relevant LCA studies for steel/iron, aluminum, copper and nickel is presented in Table 2. A nice review on the environmental impact of metal production processes written by Norgate et al.^48^ complements some of the more recent studies listed in our work.

*Iron*: Table 3 shows the theoretical CO_2_ emissions per kg of produced iron linked to reducing strategies that can be implemented for the primary production of iron. It includes the use of metallurgical coke in blast furnaces which is converted into CO for the reduction, the use of natural gas which is reformed into H_2_ and CO for the direct reduction of iron oxide pellets, the use of hydrogen for the direct reduction as well and finally the use of electrons in molten oxide electrolysis processes. This table confirms that direct CO_2_ emissions can be completely avoided if green hydrogen and electrolysis approaches are implemented in the future. It provides a good comparison basis when analyzing the data that are presented in this section.

**Table 3:** Theoretical direct CO_2_ emissions per kg of produced iron for different reducing agents.

Reducing agent	Main reaction	CO_2_ emissions (kg/kg Fe)
Coke (CO)	3CO(g) + Fe_2_O_3_(s,l) $\rightarrow {}$ 2Fe(l) + 3CO_2_(g)	1.18
CH_4_ (CO/H_2_)	2H_2_(g)+CO(g)+Fe_2_O_3_ $\rightarrow {}$ 2Fe(s) + 2H_2_O(g) +CO_2_(g)	0.39
H_2_	3 H_2_(g)+Fe_2_O_3_(s) $\rightarrow {}$ 2Fe(s) + 3H_2_O(g)	0
Electron	2Fe_2_O_3_(l) $\rightarrow {}$ 4Fe(l) +3O_2_(g)	0

The environmental impacts related to the primary production of steel via the blast furnace/basic oxygen furnace (BF/BOF) or the Direct Reduction Iron/Electric Arc Furnace (DRI/EAF) routes is well documented. As such, there exists a large literature of LCA studies for both production routes. Operation of the BF/BOF has a Global Warming Potential (GWP) impact in the order of 1.59–2.46 kg CO_2_ per kg crude steel according to a select number of studies.^49–53^ For the BF/BOF route, the largest CO$_2$ emitter unit operation in the process is the energy-intensive and strongly reducing blast furnace accounting for 40–69% of total GHG emissions. The remaining contributions are split between coke ovens, sintering plants and BOF. For the electric arc furnace path , LCA studies suggest a GWP impact in the order of 0.86–0.91 kg CO_2_/kg crude steel.^49,51^ Indirect emissions are due to the consumption of fossil fuels for electricity and direct emissions are caused by the consumption of graphite anodes during operation of the EAF. It is worth noting the scrap ratio in the feed sent to the EAF is an important contributing factor to GWP^69^ as this stream is already in its metallic state and does not need to be pre-reduced in a DRI furnace. In developed countries, the feed can reach up to 100% scrap while in other countries the ratio is closer to 75%.^70^

*Aluminum*: Cradle-to-gate LCA of primary aluminum production^48,54–57^ considers the mining of bauxite, alumina production (Bayer process), smelting (Hall–Héroult electrolysis) and casting. In a critical review of aluminum LCA, Lui^71^ identified some shortcomings of LCA on aluminum production; notably the difficulty of a complete cradle-to-grave LCA, the use of generic industry-wide data, the challenge of secondary production allocation and the focus on GHG emissions and energy consumption. Nonetheless, such studies are useful to identify areas in the process for potential improvements with the perspective of integrating greener reactants and more renewable energies in this industry. In Lui’s review of LCA, typical values of 9.7–18.3 kg CO_2_/kg Al ingot were found for the primary production of Al. The higher values are attributed to processes operated in regions with a high reliance on fossil fuels to produce electricity (such as coal power plants in China). A LCA made using data from China calculated that electrolysis was responsible for 63.3% of total GHG in the primary production process followed by alumina production at 18.7%, carbon anode consumption at 8% and bauxite mining at 7.4%.^57^ More specifically, smelting operations actually require between 13^72^ and 14 kWh^73^ per kg of Al. Depending on the reliance on thermal power of an electricity grid, the actual energy demand may be higher as presented in Table 2. The calcining of the hydrated alumina obtained from the Bayer process explains the important energy requirement of extraction operations which ranges between 5.8 kWh^73^ and 8.5 kWh^74^ per kg of Al. Studies on aluminum recycling^57–59,71^ confirmed the considerably lower impacts of recycling with a typical range of 0.3–0.7 kg CO_2_/kg Al ingot. Again, the higher values are from refineries relying on fossil fuels. The main contributor is the remelting and casting accounting for about half of secondary production GHG emissions. Scrap collection and pre-treatment each account for a quarter of GHG emissions. Finally, it has to be pointed out that the study of Norgate et al.^48^ suggests a staggering cradle-to-gate energy requirement of 58.6 kWh per kg of Al, which is on the higher end of the spectrum considering the individual contributions found in the literature and reported in the table.

*Ferronickel alloys*: As stated previously, they are typically produced from lateritic ores through a pyrometallurgical process that couples the use of rotary-kilns to electric arc furnaces (RKEF). Cradle-to-gate LCA of nickel production,^60–63,75^ suggests energy demands of 30.6-44.28 kWh/kg FeNi and climate change impacts of 6–12.60 kg CO_2_/kg FeNi as reported by Wei^62^ in his review. In one LCA,^60^ the smelting process accounted for 52.5% of energy demands and 56.18% of global warming potential. In particular, the consumption of carbon electrodes and paste in the electric arc furnaces is the main contributor to direct CO_2_ emissions. Refining operations account for 23.5% of energy demand and 22.90% of GWP due to electrical consumption.

*Nickel*: Pure nickel can be produced from sulfide ores through a common pyrometallurgical path that includes mining, concentration, melting in a flash smelter to obtain a nickel-rich matte that is then refined to produce nickel metal.^76,77^ From cradle-to-gate LCA,^19,61,62,64^ the global warming potential fits in a range of 7.64–14 kg CO_2_/kg Ni. Smelting and refining were identified as the main contributors (around 60% of total emissions) due to electricity and fossil fuel consumption during these steps. It is worth noting that the production of nickel metal is also possible through hydrometallurgical processing of lateritic ore via pressure acid leaching, solvent extraction and electrowinning.^78^ This route involves substantially higher energy demands, in the order of 53.9 kWh per kg of Ni according to Norgate and Jahanshahi^48^ and Rankin.^74^ To complement our analysis, one has to remember that the demand for nickel should drastically increase in the future mostly because of car electrification which will require large quantities of it for the battery production.^79^ Given that nickel from sulfide deposits will not suffice, the green industry will need to purchase nickel with high CO_2_ footprint; the RKEF route to ultimately produce pure nickel requires about 125.8 kWh along with CO_2_ emissions of about 24.9 kg per kg of Ni (Sorowako data reported by Mudd^80^).

*Copper*: LCA studies^65–68^ considering the boundaries of the cradle-to-gate of the production of Cu evaluated the main environmental impacts related to different processes. As presented in Table 2, the studies from different regions focus on the assessment of impact indicators as cumulative energy demand and global warming potential. The required energy for primary copper varies between 9.02 and 46.7 kWh/kg Cu with a GWP reported in a range of 1.91–6.0 kg CO_2_/kg Cu. For recycled copper these values are unsurprisingly lower. Both parameters are ultrasensitive to the source of the grid-based electricity production.^66^ According to Sanjuan et al.,^68^ materials, energy and emissions streams related to copper production also depend in part on geology characteristics of copper deposits. Besides that, the type of refining process is also a relevant aspect that must be considered. In a thorough and meticulous study, Coursol et al.^81^ calculated the energy consumption for Noranda/Teriente bath smelting under various conditions with values ranging from 3.16 to 3.53 kWh/kg (11.363–12.708 MJ/kg).

These LCA studies presented in this section are useful to identify areas of potential improvement as they provide quantitative impact figures down to specific steps in each primary metal production process. Nonetheless, such studies have limitations. Most of them focus on current proven technologies as the required inventory data are readily available. In contrast, there are few studies involving emerging technologies. Even so, a variation in results is possible from using different software and/or databases and is a challenge when comparing LCA studies.^68^ The geographic scope is another limitation as most studies use data from Europe or China. This mostly affects how electricity is accounted for due to the composition of energy grids with regards to fossil fuels and renewables.

## Greener reactants and recycled feeds in pyrometallurgy

The primary production of metals from oxides and sulfides has been historically dominated by the use of carbon and air which are cheap and naturally abundant reactants. Moving away from these resources also implies the necessity to produce synthetic reactants such as bio-fuels, hydrogen, ammonia and pure O_2_. These synthetic chemicals come with a high energy price tag: green hydrogen produced via water electrolysis requires about 45 kWh per kg^82^ while ammonia obtained from a fully-green Haber–Bosch process would have an energy consumption of about 8.7 to 10.3 kWh/kg according to the design proposed by Rouwenhorst et al.^83^ This is to be compared with the electrical energy requirement of about 0.4 kWh per kg to produce iron from an electric arc furnace exclusively fed with scrap.^84^ This section introduces the greener reactants the pyrometallurgical industry is considering to use in the future.

### Greener reactants

#### Bio-fuels

Fuels extracted from biomass are called biofuels. They are attractive because they have the potential to limit or even reduce our CO_2_ burden as well as being a sustainable alternative to face the depletion of fossil fuel.^85^ Currently, biofuel sources are classified into four generations^86^: edible-based biomasss such as sugar and corn (1st generation), inedible-based lignocellulosic biomass (2nd generation), aquatic feedstock (3rd generation) and bioengineered microorganisms (4th generation). It is to be noted that the displacement of agricultural lands that could be used to feed populations is a major ethical concern which promoted the abandonment of the 1st generation of bio-fuels. As pointed out by Bright et al.,^87^ the environmental benefits of using bio-fuels must be quantified with care as they are closely related to the carbon-cycle of their source: an internal combustion engine car produces about 2.5 metric tons of CO_2_ per 10,000 km (i.e. about 250 g per km^88^), which requires the mature growth of about 10 trees in a year (i.e. 70 kg of sequestered carbon per tree in a year^89^) for the CO_2_ capture. In other words, carbon neutrality is not equal to climate neutrality. In fact, some researchers have already raised some doubts about the carbon-neutrality of some bio-sourced fuels and carbonaceous materials in pyrometallurgical applications,^90^ which puts prime importance on the parameters and methodology used in LCA studies.

#### Bio-char

Carbonaceous materials such as coal and coke are key ingredients in the extractive metallurgy of many metals and ferroalloys.^91^ They can also directly react with metals to produce carbides^92–96^ which are nowadays used in many high temperature applications. Injection of coke in slags is an efficient and cheap way of partially recovering valuable elements such as chromium during steel recycling in electric arc furnaces. Moreover, a blend of coal tar pitch and coke can be processed to produce graphite electrodes used in electrolysis cells and electric arc furnaces. In these applications, the good thermal and electrical conductivity, low thermal expansion coefficient and high thermal shock resistance make graphite a perfect material. For all these reasons, many are looking into the possibility of using bio-sourced char for these applications. As pointed out by Mathieson et al.^97^ organic waste cannot be directly used in pyrometallurgical operations as either a fuel or a reducing agent because of its moisture content, its low carbon content and low calorific value. Because of that, biomass needs to be pyrolyzed first. There are four classes of biomass pyrolysis methods as reported by Ghodake et al.^98^: i.e. slow, flash, fast and gasification. According to the same authors, the production of bio-char typically requires slow heating rates (5–7 °C per min) and a specific temperature range (300–800 °C) to obtain stable carbonaceous solid biochar materials. A wide variety of organic resources can be used as the feed-stock of bio-char. Among them, animal manure (cow, pig, yak), agricultural and forestry waste (rice husk, cotton stem, walnut shell, wheat straw, eucalyptus sawdust, peach branch, wood sawdust, poplar wood, switchgrass) and sewage sludge are the most popular feed-stock in current research.^99–101^ Although chemical equivalence to coke and other carbonaceous products is technically achievable, one major limitation of bio-char is its incompatible physical properties such as its high reactivity and low mechanical strength when compared to fossil coke (Table 4). Additionally, bio-chars tend to contain higher contents of alkali-metal-based ashes (when compared to coke) which are to be avoided in blast furnaces^102^ as they accumulate in these reactors and attack the refractories. The bio-carbon reactivity towards CO_2_ (i.e. CO_2_ gasification of fixed carbon included in bio-carbon) is one of the most important properties of carbon-based materials for the specific metallurgical process to optimize the reduction process.^103,104^ In the iron production process, the CO produced from the heterogeneous reaction between carbon reductant and CO_2_ will react with the iron ore to produce iron.^105^ Ye et al.^106^ presented a review on the potential applications of biochar in ferrous metallurgy which include coking, iron ore sintering, metallized pellet production, BF ironmaking and EAF steelmaking. Another important field of application of bio-sourced carbonaceous materials is the production of graphite electrodes. The exclusive use of bio-sourced material precursors for these applications is currently impractical. Here is a summary of the main applications that are considered for the use of bio-char materials in pyrometallurgy:

**Table 4:** Specific properties of biomass, charcoal and fossil fuel.^107–127^

Material	Fixed carbon	Bulk density	Surface area or porosity	Crushing index	Moisture content	Energy density	Electrical conductivity	CO_2_ reactivity
	%	kg m$^{-3}$	^a^m$^2$ g$^{-1}$ or ^b^ %	–	^c^ w.b.%	MJ kg$^{-1}$	$\upmu$S cm$^{-1}$	–
Wood	15–20	400–865	55.4^b^ (oak)		12	17	Very low	High
Charcoal	65–85	700	$\ge$300^a^ (wheat residue)		5–7	29	Low	Medium-high
Coal	34–75	600-800	2–17^b^	1.63 ($\ge$ 80 mm) & 1.82 (80–60 mm)	45–63	25–30	Low	Low-Medium
Coke	86–88	1100–1400	45–53^b^		3–16	33	High	Low-medium
Bio-oil	$\ge$ 60	1200	Not applicable	Not applicable	25–30	18	No data	Medium
Biochar	$\ge$ 50	350–500	642^a^ (oak)		3–16	33	400–1000	High

^a^BET surface area.

^b^Volume %.

^c^w.b%: moisture content in wet basis.

*Lance injection in electric arc furnaces* Electric arc furnaces using graphite electrodes are at the heart of modern pyrometallurgical operations for the primary production and recycling of many metals such as iron (ferro-alloys), silicon, nickel and copper. They are also commonly used for the recovery of valuable metals from slags that are rich in Ni,^19^ Cu^128^ and Pb.^129^ One key application of bio-char is therefore its direct injection in the slags which are present in these furnaces. In iron and steel production, most of the feed is already metallic, either in the form of direct reduction iron pellets or scrap material. In this case, carbon is added in the charge for chemical heating and also added via injection as a slag foaming agent.^130^ In this process, carbon reacts with the FeO in the slag to generate CO bubbles that induce slag foaming. It is to be mentioned that charcoal and bio-based reductants have already been used in ferroalloy industries and silicon production.^131^ When compared to coal and coke, charcoal generates significant fine particles which are detrimental to the gas permeability in the stacks and necessitate increased off-gas processing. Pyrometallurgical treatment and the addition of binders can be used to improve its performance by engineering its properties.^132,133^ Charcoal should not be harvested from primary forest to be considered environmentally friendly.^90^

*Tuyere injection and coke substitution in blast-furnaces* While bio-carbon could theoretically replace all the fossil carbon added to a blast furnace, there are practical limitations which diminish its CO_2_ mitigation potential. Bio-carbon can be added in the coking blend, in the ore blend, as nut coke or in the fuel injection mixture.^134^ However, fuel injection via the tuyeres is considered by many as the easiest way to mitigate CO_2_ emissions via the use of bio-coal as it does not significantly affect the BF performance.^135^ In fact, it was reported that the complete substitution of coal by bio-coal for fuel injection had equal or better combustion performance than its fossil counterpart.^136^ It was estimated that a 33% reduction in CO_2_ emission could be obtained using 100% charcoal for fuel injection.^135^ In the work of Okvist and Lundgren,^135^ it was found that the substitution of 5% biocoal in briquettes and 2–5% in coke had moderate beneficial impacts on the BF CO_2_ emissions while not impairing too much the behaviour of the reactor.^135^ In another study, the successful incorporation of briquettes containing 1.8 wt% torrefied saw dust in a BF for several weeks was demonstrated. The lower carbon consumption was attributed in this case to an improved gas utilization.^137^ Coke is difficult to replace since it has good load bearing capacity; biocoal lower mechanical properties and high porosity and reactivity affect negatively the performance of the stacks in the blast furnace. However, there is a potential to use it for smaller furnaces which do rely heavily on the coke mechanical properties. A successful example would be the Brazilian mini blast furnaces with a productivity of 1000 tonnes of hot metal per day. They are presently operated exclusively with charcoal produced from eucalyptus wood.^138^ Although their net productivity is an order of magnitude smaller than typical blast furnaces they offer an alternate solution to mitigate primary steel net CO_2_ emissions.

*Carbon material precursors for graphite anodes and ramming paste* Primary aluminum production via the traditional Hall–Héroult process consumes around 0.4–0.46 kg of C/kg of Al (which directly depends on the current efficiency of the cell).^139^ Most of this carbon is being used by the main anodic reaction of the cell in which carbon reacts with oxygen anions available in the electrolyte to produce gaseous CO_2_. Anodes are made from calcined petroleum coke mixed with coal tar pitch and recycled anode butts. Opportunity to replace these fossil carbon sources by bio-coal and bio-tar can directly reduce the CO_2_ footprint of the aluminum industry. However, it was found that the substitution of coke by bio-coal in the anode is difficult since it increases the anode’s CO_2_ reactivity and resistivity while it decreases its density and mechanical strength. Those drawbacks are linked to its increased porosity, lack of sulfur and greater presence of hetero-elements.^133^ Presently, standard anode properties are kept when at most 3 wt% bio-coal are used.^133,140^ The physico-chemical properties of biocoal also prevent its proper surface wetting by the softened tar, which ultimately decreases the performance of the baked anodes. There are other possibilities to increase the use of bio-coal and bio-tar in anode production^141^; the most realistic approaches being the replacement of fossil binder material by a bio-based binder and the partial substitution of coke in the formulation of the anode paste. The replacement of coal tar pitch by bio-tar was suggested by enriching it with quinoline-insoluble content coming from bio-char.^132^ Finally, bio-char can be used in the formulation of eco-friendly ramming paste for Hall-Héroult pots.^142^

#### Hydrogen

Hydrogen has been identified by many scientists as the energetic vector of the future to replace fossil fuel for sustaining the development of the earth’s growing population.^143^ It has a higher heating value of 141.9 kJ/g which is about three times larger than those of methane (55.5 kJ/g) and gasoline (47 kJ/g).^144^ This makes hydrogen an attractive fuel option for transport applications. In fact, it is viewed not only as a potential fuel for cars, buses and trucks but also as an energy carrier (to power generating stations) and storage medium (when renewable energies that directly produce electrical work are available). In pyrometallurgy, it will most probably play a critical role as a reducing agent for the production of many metals as will be explained in “H_2_ reduction” section. Currently, hydrogen production solely relies on its commercial demand from the industry. Most of this hydrogen is being used for ammonia production (i.e. about 49%) followed by petroleum refining (37%) and methanol production (8%).^145^ According to the International Energy Agency (IEA), this demand has quickly grown from 18.2 Mt in 1975 to 74 Mt in 2018.^146^ As a direct consequence of all these potential application opportunities for the future, the global demand for hydrogen is forecast to increase drastically. Depending on the predicting approach, it is expected that the demand will grow from about 74 Mt up to 287 Mt in 2050 when considering a sustainable growth scenario,^143^ while the demand could reach a staggering 568 Mt by 2050 according to the most H_2_ intensive use scenario.^147^

To sustain this growth, large-scale hydrogen production strategies need to be deployed. The actual precursors for hydrogen production are natural gas (48%), oil (30%), coal (18%) and water (4% via electrolysis).^148^ This shows that the current hydrogen production routes are also large CO_2_ emitters since they are mostly sourced from fossil fuels; this type of hydrogen is called gray hydrogen. Blue hydrogen refers to the hydrogen obtained from this production route into which carbon capture and sequestration units are integrated to lower the CO_2_ emissions. Finally, green hydrogen is made from water electrolysis using a renewable energy source (such as hydropower). The theoretical energy requirement to produce 1 m^3^ of H_2_ according to Faraday’s law is $W_{t}= 2.94\, \text{kWh/m}^{3}$ compared to the practical energy consumption $W_{p}=4.78\, \text{kWh/m}^{3}$.^149^ This gap in terms of energy efficiency is a direct consequence of high overpotentials and large ohmic voltage drop leading to significantly larger applied voltages ($V_{p}$ = 1.8–2.6 V vs $V_{\text{th}}=1.23V$).

**Figure 1:**
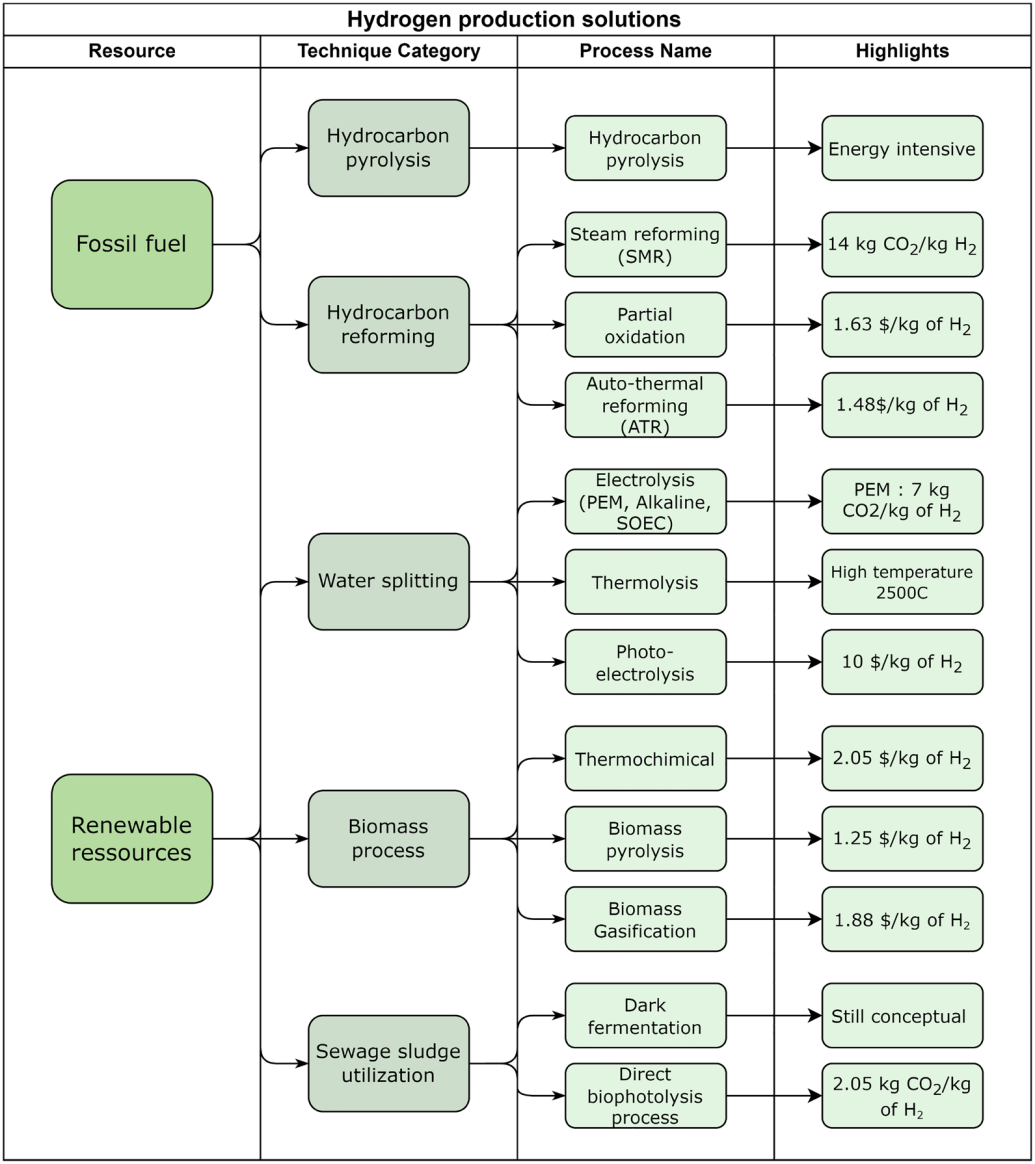
Hydrogen production paths from fossil fuel and renewable energy sources. Inspired by the work of Nazir et al.,^150^ and Hosseini et al.^151^

Figure 1 inspired by the work of Nazir et al.^150^ and Hosseini et al.^151^ summarizes all processes explored to produce hydrogen. As stated previously, fossil fuel is the most common source of hydrogen used for its production at the moment. Owing to its high efficiency (i.e. 70–85%), the Steam Methane Reforming (SMR) process is the most popular large-scale approach^150^ as well as the most polluting one (it emits 14 kg of CO_2_ per kg of H_2_ without considering CCS). It is governed by the following reaction performed between 800 and 1000 $^\circ$C:

1 $$ \begin{aligned}{\text{CH}}_{4} {(g)} + {\text{H}}_{2} {\text{O}}{(g)} \rightarrow {\text{CO}}{(g)}+3{\text{H}}_{2}{(g)}\end{aligned}$$

It is also possible to use dry reforming to produce hydrogen via the following reaction in the presence of catalysts such as rhodium, palladium or nickel^152^:

2 $$\begin{aligned}{ \text {CH}_4}_{(g)} + {\text {CO}_2}_{(g)} \rightarrow {} 2 {\text {CO}}_{(g)}+2{\text {H}_2}_{(g)} \end{aligned}$$

Methane cracking (also called pyrolysis) is another interesting and technologically simple process as it leads to the production of H_2_ without any GHG emissions (solid carbon is formed instead). Its main drawback is its high temperature required (around 1300 °C) to ensure full conversion.^145^ Water splitting processes virtually avoid GHG emissions if they are powered by renewable energies. They include water thermolysis and electrolysis. Water thermolysis involves the thermal splitting of water molecules into pure oxygen and pure hydrogen at high temperature (about 2900 K to reach a reasonable degree of dissociation).^152^ A more promising approach is water electrolysis which involves low-temperature anodic oxygen evolution and cathodic hydrogen evolution.^153^ The slow kinetics of the anodic reaction is the main barrier to increase the energetic efficiency of this process.^154^ Finally, hydrogen production via biomass or sewage sludge can be divided into two categories: biological and thermochemical processes. Biological processes involve photo-fermentation and dark-fermentation (as well as a mix of both). Thermochemical processes include pyrolysis, gasification and supercritical gasification.^155^ The very low H_2_ yield of these approaches makes them unrealistic solutions for massive hydrogen production in the future. Finally, it is to be remembered that even though hydrogen is seen as a critical synthetic chemical in the future, its use and integration into our societies come with many challenges linked to its safe storage and transportation.^156^

#### Ammonia

The large/industrial scale deployment of hydrogen faces many challenges associated with its transportation, distribution and storage. Because of that, the use of ammonia as an energy vector is considered.^157^ The production of green ammonia involves the production of green hydrogen (via water electrolysis) as well as the extraction of N_2_ from air (via an air separation unit).^158^ Pure H_2_ and N_2_ are then injected in a multi-pass Haber–Bosch reactor which operates between 400 and 500 °C at pressures between 100 and 300 bar. Single-pass processes originally operating at even higher pressure (such as the Claude process) are also being revisited because of the possibility to lower operating temperature and pressure with the use of next-generation Ruthenium-based catalysts.^159^

### Recycled materials as alternative feed to ores

The constantly increasing demand of industrial metals required for the development of technologies such as cars, planes, boats, batteries, computers and cell phones exerted a high pressure on the availability of high grade ores over the last century. The extraction of these valuable metals from rocks also came at a high energy cost along with significant environmental impact for our planet. At the end of their useful life, these technologies become significant sources of metals which need to be valorized. Countries having abundant mineral resources such as Canada have integrated waste streams in primary metal processes for decades^160^: electronic scrap is rich in copper and has been integrated into the primary production of copper from sulfides (i.e. the Noranda process); steel scrap is an important source of iron which is melted along with DRI pellets in electric arc furnaces at ArcelorMital in Contrecoeur to produce various steel products while conventional accumulators of gasoline cars are recycled to recover lead in KIVCET reactor (along with lead sulfide concentrates) by Teck at Trail Operation. Other valuable metals are also being recovered from other waste streams via pyrometallurgical operations: this includes cadmium and nickel recovered from rechargeable batteries via smelting processes,^161^ aluminum recovery from cans and aluminum foil via remelting in rotary furnaces and many other valuable metals (such as Cr,^162^ Cu,^163^ Ni^164^) from slag carbo-reduction in high-temperature furnaces among others. Important challenges associated with the integration of these streams into conventional primary metal processes include the presence of polymers which may release toxic emissions at high temperature upon their combustion and thermal degradation (such as metallic bromide release upon heating electronic waste^165^), the simultaneous presence of many metals (both noble and reactive metals) which need to be chemically separated^166^ and the contamination by oil, paint and organic waste which may promote the unwanted presence of volatile elements such as hydrogen and sulfur along with the possible formation of carbides.

There are three basic operations for the separation of valuable metals from impurities during recycling operations: i.e., oxidation, reduction and evaporation/condensation. Oxidation and reduction typically lead to the formation of slag and liquid metallic solutions while evaporation involves the transfer of elements in the gas phase. A thorough thermodynamic analysis of the potential partitioning of different impurities present in industrial metals (i.e. aluminum, iron, zinc, lead and magnesium) upon these three separation strategies was reported by Hiraki et al.^167^ In a subsequent work, Nakajimi et al.^168^ highlighted the difficulties of removing noble impurities such as copper from aluminum and iron scraps. The need to lower the concentration of Mn by dilution when recycling 3000 series alloys was also discussed in another work dedicated to aluminum recycling.^169^ This section reviews the opportunities of using metallic-based waste streams as greener sources of metals. It also covers the pyrometallurgical strategies envisaged to recover the metal fraction of these wastes.

#### Iron and steel scrap

Steel scrap is a major feed material that has been traditionally used in the steelmaking industry for a long time. It can be added to basic oxygen furnaces to adjust the heat balance during the carbon and impurity oxidation (as all these reactions are strongly exothermic). It can also be directly introduced in electric arc furnaces. Some plants can use up to virtually 100% of mixed scrap to produce their new steel from such furnaces. To do so, a tight control of the chemistry of the scrap used in the process is required. In recent years, the unwanted presence of high level of copper and zinc has become an issue. Copper may be intentionally added to steel in some specific applications, as a secondary alloying element. It is proven to increase the metal’s corrosion resistance and mechanical strength via precipitation hardening of Cu-rich precipitates.^170,171^ When it comes to applications requiring thermo-mechanical processing, Cu is an impurity that must be removed as it substantially decreases the resistance to hot rolling and leads to surface hot shortness.^172^ This is caused by the preferential oxidation of iron and the resulting formation of Cu-rich regions which are melting during hot working.^173^ Copper contamination in iron scrap usually originates from end-of-life vehicles and home electronic appliances (Cu is a common metal used for the production of electrical wires).^174^ The presence of zinc in steel scrap originates from the galvanization process used to protect its surface from corrosion.^175^ As of now, copper is only partially removed from steel scrap through shredding and subsequent magnetic separation. The inability to efficiently remove copper upon recycling leads to a long-term increase of the copper content of recycled steel.^176^ When the Cu content reaches 0.2 wt%, it causes hot shortness upon hot working.^177^ Copper, a relatively noble metal, cannot be removed by oxidation because of its low oxygen affinity. This contaminant therefore cannot be removed through common steelmaking processes.^178^ The only option in this case is to use a dilution effect, i.e. to dilute Cu in steel using fresh primary iron. Contrary to copper, zinc can be removed prior to steel recycling using hydrometallurgical and vacuum heating strategies. As for copper, the oxidation process at high temperature cannot decrease its concentration in liquid iron. According to the literature,^172^ a strategy based on the use of chlorine gas can be an option to remove copper in the form of volatile chlorides above 1000 K. In this process, oxygen is simultaneously injected in the reactor to preferentially oxidize the surface of iron particles which limits the iron chlorination and its loss.

#### Aluminum scrap

As highlighted by many authors,^179–181^ the simple remelting of aluminum and its alloys in reverberatory or rotary furnaces requires only 5% of the energy need of the primary extraction from the Hall–Héroult process.^182^ There are many unwanted elements upon aluminum re-melting such as Cu, Mn, Fe, as well as C and H (which are introduced via organic waste). These elements are integrated into the recycling loops because of poor sorting. Contamination of Al scrap by iron and organic waste is highly detrimental: iron is virtually insoluble in the aluminum matrix and forms intermetallics such as the Al_13_Fe_4_ phase upon solidification while organic waste reacts with aluminum to form carbides which are hard and brittle.^179^ Reaction of liquid aluminum with moisture is to be avoided as well since it leads to (1) the formation of oxides (such as alumina particles) that need to be filtered and (2) the generation of hydrogen which dissolves in the molten metal and forms porosity upon solidification. As pointed out by Gaustad et al.,^181^ physical separation and efficient sorting are essential to reduce contamination and limit the amount of primary aluminum to be used to dilute impurities. More recently, other impurities have integrated aluminum recycling loops such as vanadium, nickel and chromium. The presence of these elements is due to the decrease of the coke quality used in the formulation of Soderberg prebaked anodes combined with the impossibility to oxidize them during further recycling treatments.

Because of the challenges associated with impurities, the quality of the scrap is an important aspect of the recycling process. Recycling to produce wrought alloys is only feasible with clean, unoxidized and uncoated scrap of ideally a single-alloy composition. This requirement limits the secondary production of wrought alloy, but the process is otherwise a straightforward and efficient remelting due to tightly controlled scrap collection as is done for aluminum can recycling.^183^ Low-quality scrap is characterized by various levels of oxidation, organic contamination and/or a mix of several alloys and potentially other materials (because of poor sorting). In this case, remelting is not sufficient and refining of the scrap is required to remove unwanted alloying elements and impurities. Chloride or fluoride fluxing is the most common refining technique to remove reactive impurities such as Ca, Na, Mg and Li. The Hoopes process conventionally used for aluminum refining is a technique which could theoretically be used for the elimination of less reactive impurities including Cr, Cu, Fe, Mn, Si and Zn.^184^ An electrolytic cell is used with a molten purified aluminum layer as the cathode and an aluminum copper layer as the anode. This process can achieve high purity aluminum but at a significant energy cost. This scrap is, therefore, used to produce secondary cast alloys that have a higher tolerance for impurities.^181^ Solheim et al.^184^ recently proposed a simplified side-by-side geometry electro-refining cell to remove these impurities.

#### Electronic waste

Electronic equipments are made of multiple components such as microcontrollers, transformers, batteries, fuses, relays, switches, motors, resistors, capacitors, diodes, transistors, inductors, integrated circuits and circuit breakers. These components are made of metals and alloys (both ferrous and non-ferrous), ceramics, plastics, composites and more.^185^ The overall composition of the electronic waste is highly variable: Senophiyah et al.^186^ reported e-waste streams with high levels of ferrous materials (36%), lead glass (19%), brominated plastics (18%), aluminum (5%) and copper (4%) while other authors focusing on the recycling of more specific e-waste (such as printed circuit boards) reported significant amounts of ceramics (SiO_2_ and Al_2_O_3_ which compose glass fibers) along with higher amounts of copper (up to 27 wt%). Many other valuable metals (such as Au, Ag, Pd, Pt and Co) are also available in this type of e-waste.^185^ The high heterogeneity and chemical fluctuation of this electronic waste is a recycling challenge by itself, especially if one wishes to recover each individual valuable metal. We have recently reviewed all pyrometallurgical processes which are currently being explored for the recycling of the metallic fraction of these electronic waste materials.^187^ Pyrometallurgical recycling strategies include direct integration of electronic waste streams in primary metal smelting operations of copper (such as in the Noranda smelter used to produce copper from copper sulfide concentrates) and lead (such as in Kaldo furnaces used by Boliden to produce lead from Pb-concentrates). Dedicated secondary copper treatment reactors can also be used such as the Outotec Ausmelt process.^188^ Important considerations such as (1) a lower liquid phase fraction generation (at the conventional operating temperature of the smelter), (2) the presence of polymers and flame retardants which are oxidized/burnt during the air injection releasing hazardous/toxic emissions (such as dioxin, furan, bromides) that need to be captured with sophisticated gas handling systems, and (3) the substantially higher amount of released gases and heat limiting the fraction of e-waste that can be integrated into primary processes such as primary Cu-smelters. The following overall chemical reaction compares the oxidation of copper concentrate and a simplified e-waste stream in a converter:

Concentrate-CuFeS_2_ oxidation ($\Delta$H = − 2.4kJ/gram):

3 $$\begin{aligned} {\begin{matrix} &{} 6\text {CuFeS}_2(s,25^\circ \text {C}) + 13\text {O}_2(g,25^\circ \text {C}) \\ &{} \rightarrow {} 2\text {Fe}_3\text {O}_4(s,1200^\circ \text {C})+3\text {Cu}_2\text {S}(l,1200^\circ \text {C})+9\text {SO}_2(g,1200^\circ \text {C}) \end{matrix}} \end{aligned}$$

e-waste oxidation ($\Delta$H* = − 8.5kJ/gram):

4 $$\begin{aligned} {\begin{matrix} &{} x\text {C}(\text {char},25^\circ \text {C}) + y\text {Cu}(s,25^\circ \text {C}) + z\text {SiO}_2(s,25^\circ \text {C}) +{x}\text {O}_2(g,25^\circ \text {C}) \\ &{} \rightarrow {} x\text {CO}_2(g,1200^\circ \text {C})+y\text {Cu}(l,1200^\circ \text {C})+ z\text {SiO}_2(s,1200^\circ \text  {C}) \end{matrix}} \end{aligned}$$

Magnetite produced during the oxidation of the concentrate (reaction 3) is fluxed by SiO_2_ and will impact the energy balance. $\Delta$H* of reaction 4 was evaluated considering an e-waste with the simplified composition (wt): 0.33Cu-0.33SiO_2_-0.33C. Finally, it is to be mentioned that the design of a complete and green recycling process for the maximization of the individual recovery of all valuable elements would include efficient sorting and pre-treatment steps, low temperature calcination/pyrolysis operations to volatilize and remove organic waste and polymers, smelting operations (coupled with gas scrubbing to capture hazardous gaseous species and dust) and further post-processing of the solid/solidified residues via hydrometallurgical operations.

#### Li-ion batteries

The replacement of conventional cars by electric vehicles powered by high volumetric energy density Li-ion batteries (which are also used in other devices such as laptops and cell phones) is seen as one of the most significant efforts to lower anthropogenic CO_2_ emissions in the near future. The European Commission and Joint Research Centre^189^ estimated that 900 million electric cars would be manufactured by 2048. Different cathodic materials have been developed for these batteries such as^190^: LiCoO_2_, LiMn_2_O_4_ or LiFePO_4_ as well as Lithium Nickel Manganese Cobalt (LiNi_x_Mn_y_Co$_{1-x-y}$O_2_) and Lithium Nickel Cobalt Aluminum Oxide (LiNi$_{0.8}$Co$_{0.15}$Al$_{0.05}$O_2_). According to the recent technico-economical analysis of Muralidharan et al.,^191^ the most expensive reactant in the synthesis of these cathode materials is cobalt followed by nickel and copper. These metals therefore should be recovered from end-of-life batteries as they are highly valuable. An in-depth literature review on Li-ion battery recycling via pyrometallurgical processes was recently performed by Makuza et al.^192^ One possible option is the use of these end-of-life batteries as secondary feedstock material in the primary extractive metallurgy of cobalt, nickel and copper process routes, which is actually done by companies such as Glencore(Xstrata).^193^ Others are also looking into the possibility of injecting these waste materials along with sulfide concentrates directly in nickel flash furnaces or to alternatively integrate them into the slag generated from this process for subsequent metal recovery via slag reduction using reducing agents such as methane.^194^ This research strategy will not necessarily recover lithium, as it may be lost in the slag or gas phase. Efforts are being deployed to design custom recycling routes for the recovery of lithium. They mostly involve leaching in sulfuric-based aqueous solution and precipitation of lithium in the form of carbonate (Li_2_CO_3_).

### Alternatives to carbon-based reduction processes

The decarbonization of the pyrometallurgical industry is one of the most critical actions to mitigate climate change.^195^ Muslemani et al.^196^ presented actions and strategies that the steel industry could take in the future to lower their use/dependence on carbon-based materials. It implies (1) carbon capture, utilization and storage (CCUS) strategies, (2) the use of hydrogen and biomass as reducing agents and (3) the development of high-temperature electrolysis technologies using a molten oxide electrolyte and inert anodes. These strategies are not fully mature and are still at different levels of technology readiness. The difficulty to remove carbon-based materials in pyrometallurgy is explained as follows: thanks to the Boudouard reaction and the positive variation of entropy of the following reaction in standard conditions:

5 $$\begin{aligned} 2\text {C}(s) + \text {O}_2(g) \rightarrow {} 2\text {CO}(g) \quad \Delta S^\circ (800^\circ \text {C},1\,{\text{atm}}) = +175.6 \frac{\text {J}}{\text {mol}\cdot \text {K}} \end{aligned}$$

it is always possible to define a temperature above which it is thermodynamically possible to reduce virtually any metallic oxide into its metal state at atmospheric pressure. The carbothermic reduction is commonly used for the primary production of metals such as iron and silicon as well as many ferro-alloys (such as ferro-chromium, ferro-manganese and ferro-silicon) using electric arc furnaces.^91^ Legemza et al.^197^ reviewed the pyrometallurgical processes that use carbonaceous materials. They also listed the alternative fuels that could be used such as plastics, rubber and biomass. In some cases, the use of carbon (stoichiometric amount or excess) is undesirable as it leads to the formation of carbides. This explains the difficulty in producing aluminum (formation of Al_4_C_3_) and titanium (formation of TiC) via carbo-reduction processes. As will be shown in “H_2_ reduction” section, other approaches such as metallothermic reduction are required in this case. The following section presents alternatives to conventional carbo-reduction processes.

**Figure 2:**
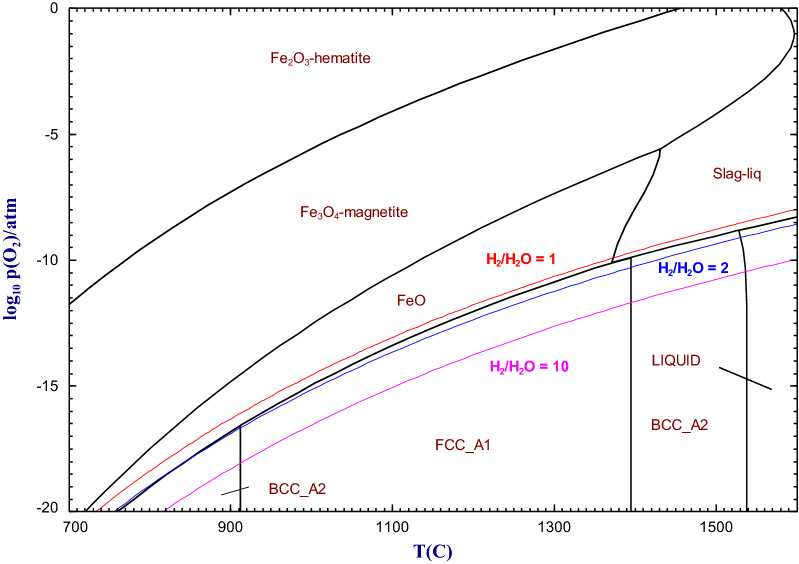
Fe-O predominance diagram as calculated by the FactSage software.

#### H_2_ reduction

Hydrogen has the ability to reduce oxides under low partial pressure of H$_2$O. Unlike the reducing conditions imposed by the presence of an excess of solid carbon in a system, it is difficult to impose the partial pressure of oxygen when using H$_2$(g). This is caused by the extra degree of freedom associated with the following reaction:

6 $$\begin{aligned} 2\text {H}_2(g) + \text {O}_2(g) \rightarrow {} 2\text {H}_2\text {O}(g) \end{aligned}$$

According to Eq. (6), the $\frac{P_{\text {H}_2}}{P_{\text {H}_2\text {O}}}$ ratio will have to be carefully imposed to the system to reduce and prevent re-oxidation of the produced metal. Figure 2 presents the calculated Fe-O predominance diagram. It shows how the H$_2$/H$_2$O ratio impacts the possibility to produce metallic iron as a function of temperature. For instance, an H$_2$/H$_2$O molar ratio of 1 is not sufficient to ensure a complete reduction, while a large H$_2$ excess induced by a ratio of 10 allows the reduction for virtually any temperature range. This section presents the recent developments related to the use of H$_2$ as a reducing agent in pyrometallurgy.

**H**$_2$ *in Iron and steelmaking*

Significant progress to reduce direct CO$_2$ emissions associated with the primary production of iron started about 55 years ago with the development of the Midrex^198^ and HYL processes. Natural gas is currently the main reactant for the reduction in these two technologies because of its availability and cost (when compared to H$_2$). This gas is reformed to produce CO and H$_2$ either in a distinct reforming unit using Ni catalysts (Midrex) or *in-situ* (HYL) since metallic iron is a natural catalyst for the reforming reactions. While the furnaces of these two technologies could accept pure H$_2$ as the reducing gas stream, one fundamental aspect would need to be adjusted with great care, i.e. the energy balance inside the reactor. The following individual reactions are taking place inside DR furnaces when natural gas is used:

7 $$\begin{aligned}&3\text {H}_2(g) + \text {Fe}_2\text {O}_3(s) \rightarrow {} 2\text {Fe}(s) + 3\text {H}_2\text {O}(g); \quad \Delta H^\circ (1000^\circ \text {C})=+61.4 \text {kJ} \end{aligned}$$

8 $$\begin{aligned}&3\text {CO}(g) + \text {Fe}_2\text {O}_3(s) \rightarrow {} 2\text {Fe}(s) + 3\text {CO}_2(g); \quad \Delta H^\circ (1000^\circ \text {C})=-35 \text {kJ} \end{aligned}$$

**Figure 3:**
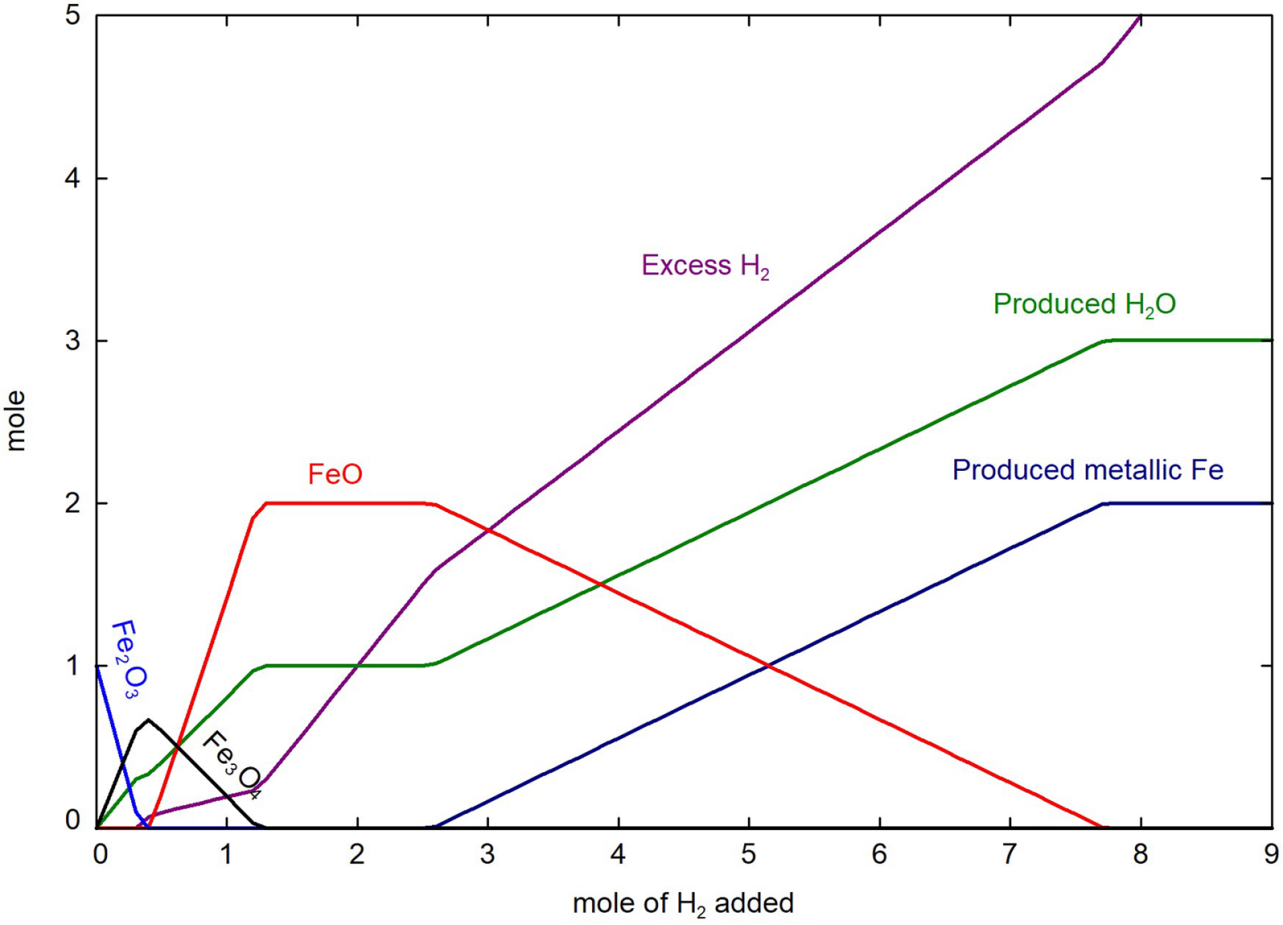
Theoretical amount (moles) of reduced Fe produced at 1000 °C as a function of the amount (moles) of H$_2$ injected in a system initially containing 1 mole of Fe$_2$O$_3$, P$_{tot}$ = 1atm.

According to Eq. (7), the reduction of hematite by H$_2$ is strongly endothermic while the reduction of hematite by CO (Eq. 8) is an exothermic process. Pre-heating or additional fuel/energy sources will therefore be required to provide this heat for the reactor to operate. Finally, it is of prime importance to mention that the stoichiometric reaction (7) cannot be targeted in a reactor as it would lead to unacceptably high water vapor pressure. Figure 3 shows the number of moles of reduced iron as a function of the amount of injected H$_2$ for a system that initially contains 1 mole of Fe$_2$O$_3$ at 1000 °C. This figure confirms that more than 8 moles of H$_2$ are theoretically required at this temperature to ensure a complete reduction of iron. As a direct consequence, the off-gas of the DRI furnace needs to be de-humidified and recycled to use the excess H$_2$ . DRI metallized pellets using H$_2$ will also lack the presence of dissolved C, which favors the melting of the metallized pellets afterwards in the electric arc furnace. Here is a list of all H$_2$-based technologies that are being considered for the primary iron production:

- Hydrogen direct reduction (H-DR) in MIDREX^29,199^ and HYL^200^
- Hydrogen flash smelting^201^
- Hydrogen-enriched blast furnace^202^
- Hydrogen plasma smelting reduction (HPSR)^203^

Today, the commercial use of green hydrogen for steel production (and other greener technologies) solely depends on its production cost which is still prohibitive. Green initiatives in the steel industry such as the ones identified in projects such as ULCOS, H2FUTURE, HYBRIT, Carbon2Chem and SALCOS^204^ are not economically viable at this time due to the competitive price of CO$$_2$$ allowance ($/kg of CO$$_2$$), even with government funding to support it. Those green initiatives will therefore depend in the future on how steel buyers (mostly from the automotive and manufacturing industries) are keen to pay a bigger price to get fossil-free steel. Several groups announced in recent years their intention to build greener cars, including Volvo in collaboration with SSAB releasing their first fossil-free steel vehicle.^205^ As a final remark regarding the use of hydrogen as a reducing agent, it should be mentioned that it is also commonly used in the steel industry to generate reducing atmosphere to prevent oxidation of the steel surface before hot-dip galvanizing operations.^206^

**H**$_2$ **reduction of other metals**

Rukini et al.^207^ listed some metal powders that are commercially produced using hydrogen reduction. It includes the production of refractory metals like tungsten and molybdenum obtained via solid-state reduction of their respective oxides. As an example, the reduction of WO$_3$ is carried out in rotary furnaces at temperatures between 600 and 1000 $^\circ$C.^207^ Other metals such as cobalt and nickel can also be produced using hydrogen reduction of intermediate compounds (mostly carbonates, sulfates and oxides) obtained from hydrometallurgical routes. It is to be mentioned that hydrogen reduction is mostly implemented for solid-state reactions for several reasons. First, it is applied to the production of refractory metals which melt at temperatures much greater than the ones required to reduce their respective oxides. Second, lower operating temperatures typically lead to energy savings and lower maintenance cost. Third, hydrogen is virtually insoluble in most solid metals. On the other hand, hydrogen solubility in liquid metals is much greater, which can lead to porosity formation upon solidification. Degassing strategies such as inert gas bubbling, vacuum heating and ultrasonic degassing can be implemented to remove dissolved hydrogen from metallic melts.^208^ In some specific applications (such as metallic foam production), the formation of a high volume fraction of porosities is intentionally induced by exploiting this large solubility difference.^209^ As for iron, reduction thermal plasma processes have also been tested for the lab-scale production of Cu, W, Co, Rh, Ge, Al, Ti, Cr, Mo, Ta, Sn, Ni and Zr.^207^ Such processes involve the generation of an hydrogen plasma which is much more energetic and less thermodynamically stable than H$_2$, leading to a much higher reducing strength.

#### Metallo-reduction

Historically, specific metals such as tantalum^210^and niobium^211^ have been extracted using metallothermic reductions performed via the use of highly reactive metals such as sodium and aluminum. Both tantalum and niobium have a strong tendency to produce stable carbides, which partly explains why they are not traditionally obtained from carbothermic reactions. Table 5 taken from the work of Jack^212^ provides a list of highly thermodynamically stable metal carbides. This table also justifies why other metals such as titanium, vanadium and tungsten are also being considered as candidates for metallothermic processes: they all form stable carbides which is highly undesirable in carbo-reduction processes.^213^ Reactive metals used as reducing agents in metallothermic reduction are calcium, sodium, magnesium and aluminum. These native metals are not available in the earth crust and need to be extracted from concentrates as well. Theoretical energy requirements for the production of these metals are presented in Table 6. H$_2$ is also presented in this table for the purpose of comparison. This table shows that sodium is theoretically the least energy expensive metal to produce for metallothermic reduction to remove oxygen from a system (379.2 kJ per mol of extracted oxygen). From a theoretical perspective, hydrogen is an interesting choice as well. However, as presented in “H_2_ reduction” section, stoichiometric reduction reactions cannot be targeted when using H$_2$ as they lead to strongly oxidizing conditions promoting re-oxidation of the metal. Because of that, hydrogen becomes in fact one of the most unattractive reducing agents from an energetic perspective.

**Table 5:** Highly thermodynamically stable refractory metal carbides, inspired by Jack.^212^

Carbide	Density (kg m$^{-3}$)	Melting temperature (°C)	$\Delta$G° (kJ/mol)
TiC	4910	3000	− 177.2
ZrC	6560	3400	− 193.2
VC	5710	2700	− 98.1
NbC	7780	3600	− 132.8
TaC	14,480	4000	− 142.7
WC	15,700	2700	− 38.3

Values of $\Delta$ G$^0$ were obtained from the FactSage software (FactPS database).

**Table 6:** Theoretical energy requirement for the production of reactive metals from their oxides as calculated using the FactSage software.

Metal	Theoretical extraction reaction	Energy requirement kWh/kg	kJ/mol of removed oxygen
*Reactive metal*			
Na	$2\text {Na}_2\text {O} \rightarrow {}4\text {Na}+\text {O}_2$	2.3	379.2
Mg	$2\text {MgO}\rightarrow {}2\text {Mg} + \text {O}_2$	6.5	569.2
Ca	$2\text {CaO}\rightarrow {}2\text {Ca} + \text {O}_2$	4.2	603.4
Al	$2\text {Al}_2\text {O}_3\rightarrow {}4\text {Al} + 3\text {O}_2$	8.1	527.4
H$_2$	$2\text {H}_2\text {O} \rightarrow {} 2\text {H}_2 + \text {O}_2$	32.7	237.2*

*Considering stoichiometric reduction reactions.

## Renewable energies in pyrometallurgy

There are clear efforts in the heavy industry to identify opportunities to lower CO$_2$ emissions of energy-intensive operations such as high temperature furnaces^214^ and heavy transportation.^215^ In fact, the literature on this subject is rich and covers many aspects linked to the quantification of various impacts of specific technologies through life cycle analysis (LCA). There are two approaches when it comes to the integration of cleaner technologies in pyrometallurgy. The first approach consists in the development and implementation of completely new unit operations or processes to replace obsolete technologies, i.e. a complete paradigm shift of technology. Electrolysis of iron in a molten oxide electrolyte^216,217^ is an example of such a potential change of paradigm in the primary production of iron. The second approach is the adoption of retrofitting strategies to modify existing processes, which limits the required investment and cost. This includes efforts in iron-ore pelletizing processes to replace fossil fuels by hydrogen^218^ or to retro-fit hot plasma torch systems^219,220^ in existing kilns. The integration of inert anodes in the production of primary aluminum lies in between these two approaches: in theory the objective would be to replace consumable graphite by inert anodes using either metals/alloys, oxides or cermets^221^ without changing the actual operating conditions of existing Hall–Héroult cells. In practice, it may be difficult to achieve this inert anode retro-fitting mostly because of the cryolite corrosiveness. One critical factor in the definition of an ideal alternative technology to an actual polluting process is the availability of renewable energy at a given location. In regions where clean power grids are accessible (see for example the province of Quebec in Canada where virtually 100$\%$ of its electricity is generated from hydropower^222^), there are many alternative options to conventional CO$_2$-emitting pyrometallurgical unit operations as it will be presented in this paper. In remote locations where power grids are not available or for countries with limited renewable energy infrastructure at this time, clean energy vectors must be used. These energy vectors include hydrogen,^223^ ammonia,^157^ accumulators/reversible batteries^224^ and even metals.^10^ Recently, the theoretical conception of dedicated clean power grids to operate entire primary metal production sectors was analyzed. Pimm et al.^225^ explored the possibility to implement a low CO$_2$-emission iron-making integrated strategy for the UK. It relies on the use of direct reduction iron reactors and electric arc furnaces. According to these authors, the entire iron production of the UK would require with their proposed approach 1.3 GW electrolysers, 3 GW of wind power, 2.5 GW of solar power, 60 MW of combined cycle gas with carbon capture, 600 GWh/600 MW of hydrogen storage, and 30 GWh/130 MW of compressed air energy storage.

### Energetic efficiency

Most renewable energy infrastructures ultimately convert available clean energy (hydro, wind, solar and geothermal)^226^ into electric work in the form of either alternative current (such as hydro-turbines and wind-turbines) or direct current (such as photo-voltaic cells and fuel cells^227^). In pyrometallurgy, most (if not all) applications require high power source (high voltage/high amperage). The main reason for this is the necessity to use large-scale reactors to lower the production cost per ton of produced metal. This is the case for typical 50 MW electric arc furnaces used in iron- and steel-making^228^ as well as for 400 kA AP40 Hall–Héroult cells used in the aluminum industry.^229^ Currently, alternative current coming from power grids is the only available option to power these large-scale industrial technologies. As mentioned previously, electric technologies typically offer greatly reduced environmental footprint if they are powered by a clean energy grid (see for example the case of water electrolysis to produce blue hydrogen^230^). Assuming the availability of a clean power grid, the energetic efficiency of a unit operation $\eta ^{\text{unit}}$ needs to be precisely evaluated if an accurate LCA analysis is to be performed on a given process. In pyrometallurgical operations, the energetic efficiency of furnaces and electrolyzers (i.e. excluding heat engines and thermo-pumps) may be simply defined as the ratio of the theoretical energy requirement $E^{\text{thermo.}}$ to the real energy ouput of the unit operation $E^{\text{real}}$, i.e.:

9 $$\begin{aligned} \eta ^{\text {unit}}=\frac{E^{\text {thermo.}}}{E^{\text {real}}} \end{aligned}$$

Laws of thermodynamics define the minimal energy requirements of a process. Computational thermochemistry packages such as FactSage are ideal tools to assess $E^{\text{thermo.}}$ for various pyrometallurgical processes.^231^ The evaluation of the energy efficiency of reheat furnaces used prior to the hot rolling of steel billets was presented for example by Si et al.^232^ It was shown that such a furnace has an efficiency of about 60% (i.e. the fraction of the energy actually used to heat the billets to the target temperature).

It is to be noted that a process may involve *i* energy conversion steps in which case an overall energetic efficiency $\eta ^{\text{overall}}$ needs to be defined:

10 $$\begin{aligned} \eta ^{\text {overall}}=\prod _{i} \eta ^{\text {unit}}_{i} \end{aligned}$$

A value of $\eta ^{\text{overall}}$ close to one implies that the maturity of the technology is high and that no further development is achievable. A low value of $\eta ^{\text{overall}}$ means on the contrary that the technology can be improved, at least from a theoretical point of view. Important kinetic limitations such as anodic and cathodic overpotentials in electrolysis technologies^233^ as well as the unavoidable presence of temperature gradients and high temperature output streams^234^ may prevent reaching a value of $\eta ^{\text {overall}}$ close to one in most pyrometallurgical processes. Strategies to harness the residual energy of these processes to increase $\eta ^{\text {overall}}$ will be presented in “Valorization of residual energy in pyrometallurgical processes” section. For pyrometallurgical processes, it includes using high-temperature flue gases of furnaces and reactors to preheat concentrates/scrap as well as other input streams or to produce steam that can be used to power a turbine or heat buildings. When comparing two zero-emission (or close-to-zero emission) technologies such as hot plasma torches and green hydrogen to replace fossil fuel in high temperature furnaces, this overall energetic efficiency should be used along with LCA results to determine which technology should be preferred.

### Heating alternatives to conventional fuel-oil burners

Burners are used in a multitude of unit operations in pyrometallurgy: they provide the energy to calcine concentrate in kilns,^235^ to melt scrap in reverberatory furnaces,^236^ and to bring extra energy in cold spots of electric arc furnaces^237^ among others. Many different types of fuels have been used throughout history: from heavy oils^238^ and exhaust gases coming from pyrometallurgical reactors (such as blast furnace exhaust gases^239^), up to clean natural gas,^240^ all available and cheap fuels have been burnt. In fact, even high-sulfur fuel oil has been used in the calcination/pre-reduction of nickel saprolite concentrates to convert nickel oxide into nickel sulfide.^241^ In conventional burner technologies, important aspects to consider when heating a system are: (1) the quantification of the energy required to heat a given mass of reactants to a desired temperature (typically expressed in $\frac{{\text {MJ}}}{{\text {kg}}}$) as well as the time it takes to reach this temperature; (2) the energy released by the combustion per unit of normalized volume ($\frac{\text {MJ}}{\text {Nm}^3}$) which is also called the higher/lower heating value; (3) the adiabatic temperature of the generated flame and its resulting heat transfer properties (both convective and radiative). In pyrometallurgical processes, both direct (flame impingement heating) and indirect heating methods are used. Glass and metal (re)-melting is often performed via the direct exposure of the charge to flames^242^ while calcination operations are typically using indirect heating to prevent ring effects (i.e. unwanted partial melting of the solid charge). Other engineering and environmental factors to consider in conventional burner technologies are the CO$_2$ and NO$_x$ emissions,^243^ as well as the wear of the corrosion materials which are used to protect the reactor.^244^ When replacing one fuel by another in a conventional burner, many engineering considerations should be taken regarding the design and material selection of its main components (which include a steel nozzle and a refractory throat or tile.^245^) The flame velocity and adiabatic temperature, as well as the amount of generated exhaust gas (which influences convective heating) are among the fundamentals aspects to consider.

#### Alternative fuels

Green power grids which can support pyrometallurgical operations are not always available. Remote location of the plant (which cannot be connected to the grid) or the non-renewable energy source used to generate the electricity at a given location are situations that prevent the use of clean electric-based heating systems. To circumvent these limitations, alternative green fuels are being considered for the replacement of fossil fuels. Examples of alternative fuels that could be used in the cement industry for the carbonate calcination step were recently presented in the literature.^246^ These alternative fuels include hydrogen, ammonia, bio-fuels and even metals.

##### Hydrogen

It is seen by many scientists as one of the most important energy vector in the future. In fact, its potential use in both domestic and industrial applications has been considered since the 1970s.^247^ As previously explained, hydrogen is not naturally available on earth and can be produced from either natural gas reforming (blue hydrogen) or via water electrolysis (green hydrogen). In the pyrometallurgical industry, its combustion in conventional burners would lead to the production of water, which can be seen as a clean chemical reaction. However, as for hydrocarbon fuels, the use of air in conventional burners will also lead to undesirable production of NO$_x$^248^ (which is exacerbated by the higher adiabatic temperature of the H$_2$ flame). This environmental problem can be lowered by using O$_2$-enriched air. The substitution of natural gas by hydrogen leads to other challenges such as flashback, potential hydrogen embrittlement of the steel components, safety, etc.

##### Ammonia

Many scientists and engineers see ammonia becoming a critical energy vector for industrial heating operations in the future. In burner technologies, the use of pure ammonia comes with many challenges^249^: it has a a low burning speed (easy to lose the flame) and it produces high amount of NO$_x$ gases. Ammonia could also be blended with natural gas to lower CO$_2$ emissions.^250^

##### Bio-fuels

As highlighted by Kohse-Hoinghaus et al.,^251^ biofuel designation encompasses a wide range of chemicals such as alcohols, esters, ethers and even nitrogenated chemicals. The chemical makeup of a specific biofuel is defined by its source and associated pyrolysis process which were reviewed in “Bio-fuels” section. Their chemistry is typically defined by their content in CSHNO, ash and alkali/alkaline earth metals. Other critical properties of liquid biofuels include their Lower Heating Value (LHV), pH, water content, density, flash point and viscosity. As a direct consequence of their distinct chemistry, their combustion also leads to a wide variety of chemical species such as PAHs, soot, carbonyl compounds as well as a plethora of other potentially toxic emissions. It is, therefore, of prime importance to explore the use of a given biofuel on a case-by-case basis. Complete substitution of fossil fuels by biofuels in burner technologies is not straightforward. Strategies to integrate biofuels include an upgrade of their fuel quality^252^ along with their co-combustion with fossil fuels to mitigate their impact. Zadmajid et al.^253^ reported that liquid biofuel can be used in swirl burners without any blending with fossil fuels (or quality upgrade) by modifying the design of the technology which is another approach for increasing the use of biofuels in pyrometallurgical operations in the future.

##### Metals

**Figure 4:**
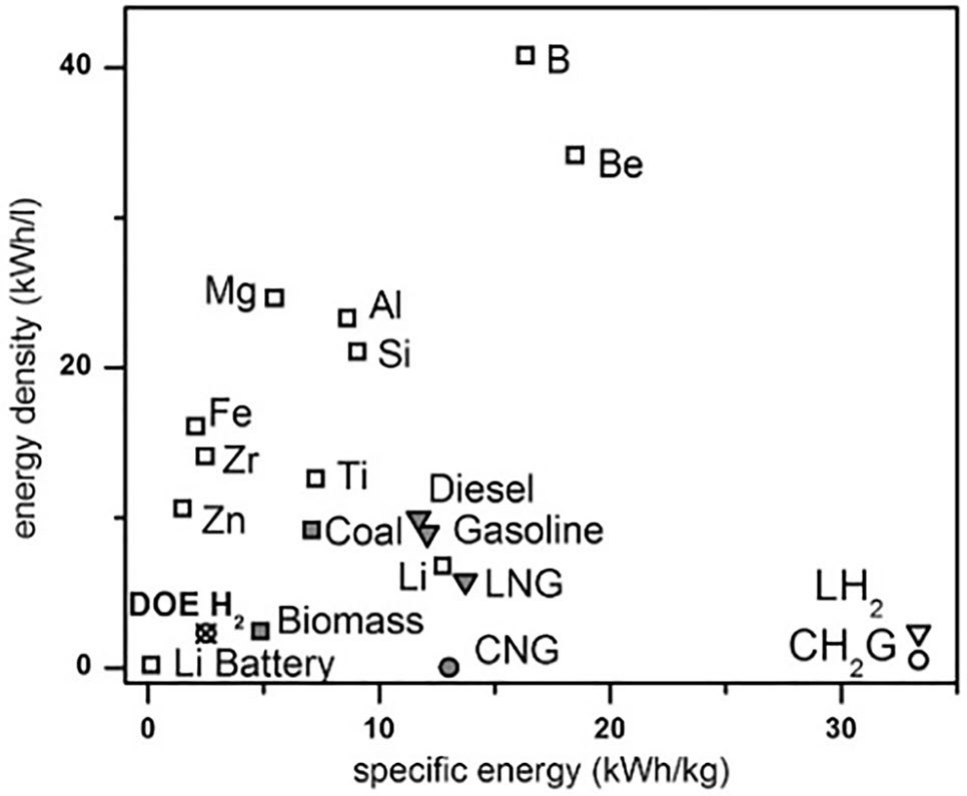
Volumetric and gravimetric energy density for various metal fuels compared to batteries, hydrogen, bio-derived fuels, and fossil fuels. Taken from Ref. 11, with permission from Elsevier.

Primary metal production is a central industrial activity required for the modernization of our societies. This explains why so many mature infrastructures are available all around the world for their production. Moreover, many green technologies are being developed for the primary production and recycling of common metals (i.e. Al and Fe) with already high TRL (ex: aluminum production using inert anodes and hydrogen-based direct reduction processes for iron production). The production of industrial metals with low CO$_2$ emissions and moderate energy requirements is almost within our reach. As presented in Fig. 4, metals can be viewed as potentially green fuels as their oxidation releases an equivalent amount of energy per unit of mass compared to conventional fuels (see for example Al and Mg compared to coal). They can also be viewed as energy carrier/vector with energy per unit of volume much greater than for liquid hydrogen (LH$_2$) and compressed hydrogen gas (CH$_2$G), even greater than for gasoline and diesel.

Bergthorson et al.^10^ explored the possibility to build low-carbon metal-fuel cycles for power generation and transportation applications. In their vision, metals become global clean energy vectors to be used in Rankine cycles, large heat engines, residential process heating units and external heat engines.^11^ The combustion products of metal combustion reactions are mostly oxides which can be recycled in primary processes. Aluminum as an energy vector was studied in more depth by Trowell et al.^254^ These authors concluded that "*the deployment of such a strategy by 2050 would require an average annual growth rate of 25% over the first 15 years as well as a roll out of appropriate burners, aluminum-water reactors and equipment to retrofit existing engines.*". Another potential use of aluminum as an energy carrier in the future is in air-metal batteries which may find applications in mobile technologies of the pyrometallurgical industry in the future.^255^

#### Electric-based heating systems

Another strategy to move away from fossil fuels is to select electric-based heating technologies. One mature technology already fully integrated in pyrometallurgy are electric arc furnaces^33^ as highlighted in “Electric arc furnaces” section. The operating parameters of EAF furnaces are now being optimized via AI and machine learning,^234^ which are other tools to reduce CO$_2$ emissions and maximize energy efficiency. Here are some other electric-based heating strategies.

##### Induction furnaces

The passage of an alternative current inside a conductive coil induces a magnetic field which in turn generates eddy/Foucault current inside the solid mass to heat/melt. These currents flowing in conductive materials (such as iron, aluminum, copper, graphite, gold and silver) heat them via the Joule effect. This heating strategy presents many advantages compared to conventional burner heating systems: (1) it has a higher energy efficiency typically well above 60%, (2) it promotes magnetic stirring when the charge is molten, (3) it is a cleaner technology as it does not generate any combustion off-gases; (4) it is a controlled and fast heating strategy. Lucia et al.^256^ reviewed the potential industrial applications of induction heating such as rail and joints hardening and aluminum foil sealing in the food industry. Many pyrometallurgical applications are targeted for the aluminum industry including remelting furnaces,^180^ semi-solid die casting^257^ and hot extrusion of aluminum billets.^258^ Induction heating is also considered in steelmaking and recycling (ex.: iron scrap cake melting^259^) and Ti alloy manufacturing.^260^ However, it is to be noted that important challenges associated with the containment of the liquid limit its integration at the industrial scale for melting applications. The refractory lining needs to be transparent to the magnetic field; an increase of its thickness will lead to leakage flux. Moreover, the refractory lining experiences extreme thermal and chemical conditions. Sharp thermal gradients, liquid metal corrosiveness and accelerated wear caused by continuous stirring are among the critical problems to be overcome in the future.^260^

##### Resistance heating

Electrical resistance furnace is the most simple electric-based heating system. It generates heat by Joule effect when passing a current in electrical resistors.^261^ As heating is a function of the power supplied, resistance furnaces ensure good control of the temperature of the melt. However, slow dynamics is a problem associated with this type of furnace^262^; it heats up much more slowly than typical fuel fired, arc melting, plasma torch and other types of furnaces. Therefore, it must be designed for a specific purpose. For instance, an electric resistance furnace using graphite heating elements is used for high purity copper production.^263^ The use of graphite rather than conventional metals or ceramic-based heating elements reduces the number of inclusions ending up in the metal.

A decantation step via an electrical resistance furnace could be added to both extractive and recycling pyrometallurgical processes to maximize the metal yield (i.e. to minimize the metal loss).^264^ When oxide mixtures are heated to a sufficiently high temperature, they start to partially conduct electricity and can therefore be heated by Joule effect^265^ This concept has been recently explored by many authors in the glass industry. This type of process is called ESR (Electro-slag remelting). The ESR process is quite complex in terms of its electrical, thermal and electrochemical characteristics, and both an electrical and ionic conductivity of slag is crucial for melting furnace design.^119^

##### Plasma torch technologies

Thermal plasma is created from the contact of plasma forming gas between two conducting electrodes. Plasma torches apply this principle to generate a continuous high temperature plasma jet. Plasma torches are advantageous due to their high energy density, maximum temperatures and heat transfer rates.^266^ From an operational perspective, plasma torches are interesting due to their small installation sizes, rapid start-up and shutdown features and use of electrical energy.^219^ This technology is associated with waste management applications but has found use in the metallurgical industry. Plasma torches are used in plasma atomization techniques to produce spherical metallic powders intended as feed stock for additive manufacturing notably for titanium.^267^ Plasma atomization generates powders with sizes of 25–250 $\upmu$m and with a higher yield of particles with lower than 45 $\upmu$m in diameter than conventional gas atomization.^268^ An interesting application of plasma torches is the production of sustainable turquoise hydrogen and carbon for use in the metallurgical industry. The electricity consumption of plasma torches is however a potential obstacle.^269^

Clean energy in electrometallurgy: Replacing chemical reactants by green electrical work is a promising way to eliminate or reduce CO$_2$ emissions related to metal making. The electrowinning of Cu, Ni, Co, Zn, Pb and other precious metals is widely done in an aqueous electrolyte while a molten salt electrolyte is used for reactive metals such as Al, Ca, Mg and Na. New electrochemical technologies are also being explored as an alternative way to extract Al, Fe and other metals.

#### Inert anodes for Al production

Some thorough reviews on the use of inert anodes for aluminum production have been performed by Deschênes-Allard,^270^ Yasinskiy et al.^271^ and He.^272^ Currently, the most widespread technology in the aluminum industry is that of prebaked carbon anodes. Thirty to forty percent of this industry’s total CO$_2$ emissions are due to the primary production of aluminum by the Hall–Héroult process,^273^ with the following overall cell reaction :

11 $$\begin{aligned} \text {Al}_2\text {O}_3(dissolved) + 3/2 \text {C}(s) \rightarrow {} 2\text {Al}(l) + 3/2 \text {CO}_2(g) \nonumber \\ \Delta G^0(960^\circ \text {C},1\text {atm}) = +690 \text {kJ} \end{aligned}$$

CO, CF$_4$, C$_2$F$_6$ and PAHs (Polycyclic Aromatic Hydrocarbon compounds) are also emitted.^274^ Although their proportion is lower, the CF$_4$ and C$_2$F$_6$ formed during the anode effect have potentials of global warming three orders of magnitude higher than that of CO$_2$.^275^ It is not straightforward to replace carbon anodes with a “green” technology that would permit the emission of O$_2$ rather than CO$_2$ according to the following reaction :

12 $$\begin{aligned} \text {Al}_2\text {O}_3(dissolved) \rightarrow {} 2\text {Al}(l) + 3/2 \text {O}_2(g) \nonumber \\ \Delta G^0(960^\circ \text {C},1\text {atm}) = +1,284 \text {kJ} \end{aligned}$$

The idea of a non-consumable anode, known as an “inert anode”, goes back as far as the invention of the Hall–Héroult process, in 1886.^276^ As early as 1889, Charles Martin Hall proposed a copper inert anode. Research continued without much success in the late 1930s with the proposal of a ceramic anode. Industrial and university research efforts have accelerated during the 1980s, in particular with Alcoa’s patented technology of a cermet anode based on nickel ferrite (NiFe$_2$O$_4$).^277^ The motivations behind these various efforts are the potential benefits of an environmental nature, of course, but also those of economic and operational nature (reduction of the frequency of anode changes, elimination of baking and anode preparation operations). Reactions (11) and (12) show that, to reduce alumina, more electrical work needs to be supplied to a cell operating with inert anodes. This is due to the exothermic oxidation of the carbon of the prebaked anodes according to reaction (11). Thus, at the same operating temperature, the use of inert anodes would require a higher potential difference of the cell, thereby increasing energy consumption.^274^ Nevertheless, inert anode technology would allow a significant increase in the lifetime of the anodes by a factor of 25 to 30, thus ensuring their economic profitability. This increase in service life would be accompanied by a reduction in the frequency of anode changes, which would stabilize the electrolytic cell in addition to reducing emissions of fluorinated substances and dust. Another advantage is that the anode effect would no longer be observed since no carbon would be available at the anode to form an insulating film of CF$_4$ or C$_2$F$_6$. In addition, the need to periodically straighten the anodes would be eliminated.^274^ One can ultimately expect a more consistent anode quality than that of carbon anodes. To be deployed, inert anode technology must meet a number of performance criteria and regulations:^278^

- Erosion rate less than 10 mm/year;
- Current density of 0.8 A/cm² with polarisation lower than 0.5 V;
- Potential drop in the anode equivalent or lower than that for carbon anodes (i.e. a good electrical conductivity is required);
- Electrical connection to simple and stable connectors;
- Mechanical robustness (i.e. withstanding vibrations, thermal shocks and stability up to 1100 °C);
- Resistance to oxidation at 1000 °C at an oxygen potential of approximately 1 atm;
- No degradation of the quality of the liquid aluminum produced (i.e. minimum dissolution of the anode in the electrolytic bath);
- Absence of Be, Cr, radioactive elements or other materials that are dangerous to health and the environment.

Cermet-type materials are hybrid between ceramic and metallic materials, and have been studied for more than 30 years as materials with a high potential to serve as an inert anode. In particular, much research effort has focused on cermets composed of NiFe$_2$O$_4$, NiO and Cu (or Cu alloys) in varying proportions. The advantages of these composites are numerous: NiFe$_2$O$_4$ is an oxide with a spinel-type crystal structure, which allows a large number of substitutions on its crystalline sub-lattices, thus modifying its properties. It is then possible to improve the stability of the anode in the cryolitic bath at high temperature and obtain a certain electrical conductivity. The addition of NiO improves the corrosion resistance of the material. Finally, Cu increases the electrical conductivity of the anode and its resistance to temperature gradients.^279^

Cassayre et al.^280^ studied the electrochemical oxidation in a cryolitic bath of Cu–Ni type metallic anodes. In a bath containing alumina, a layer of NiO is first observed at low electrochemical potential, followed by homogeneous oxidation of the alloy and detachment of the oxidized layer by increasing the potential. For Cu–Al alloys in contact with the cryolitic bath, the aluminum of the alloy oxidizes preferentially, thus forming a protective alumina film at the anode-bath interface that will ultimately break owing to the solubility of alumina in the bath. Thus, those metallic phases are not viable when exposed to the cryolitic bath under electrolysis conditions. Table 7 presents various existing inert anode materials.

To reach a sustainable technology, several issues must be taken into account in terms of development of materials, operationalization, manufacturing, cell design, etc. It is worth mentioning that Elysis, a joint venture between Alcoa and Rio Tinto, has announced the implementation of an inert anode technology by the end of 2023.

**Table 7:** Existing inert anode materials.

Material type		Anode material	Refs.
Metallic	Cu-based	Cu–Al, Cu–Mn, Cu–Fe, Cu–Ni, Cu–Ni–Al, Cu–Ni–Fe	^281–284^
	Fe-based	Fe–Ni, Fe–Ni–Al, Fe–Co–Ni, Fe–Cu–Ni	^285–287^
	Ni-based	Ni–Fe, Ni–Fe–Co, Ni–Fe–Cr	^288,289^
Oxide Ceramic	Simple Oxide	CeO$_{2}$, Cr$_{2}$O$_{3}$, CuO, NiO, SnO$_{2}$, ZnO	^290,291^
	Spinel-type	CoFe$_{2}\hbox {O}_{4}$, FeAl$_{2}\hbox {O}_{4}$, NiAl$_{2}\hbox {O}_{4}$, NiFe$_{2}\hbox {O}_{4}$, ZnFe$_{2}\hbox {O}_{4}$	^292,293^
Cermet		Al/Al$_{2}\hbox {O}_3, \hbox {Cu}_{2}$O/Cu, NiAl$_{2}\hbox {O}_{4}$/M, NiFe$_{2}\hbox {O}_{4}$/M, ZnFe$_{2}\hbox {O}_{4}$/M, WC/Ni-Fe	^294–297^

#### Alternative electrometallurgical routes

Iron oxide electrolysis in aqueous solution is currently explored as an alternative to primary Fe production. It is carried out in a concentrated NaOH solution and Fe$_2$O$_3$ is reduced at the cathode while O$_2$ is evolved at the anode. Reduction occurs directly on the solid particles either in a slurry or pressed as the cathode. The current efficiency can be higher than 95%.^298^

Another alternative is to produce iron via molten oxide electrolysis; this was only done in small laboratory scale and featured a current efficiency lower than 50% typically.^216^ The most pressing challenge related to iron oxide electrolysis is the development of a suitable inert anode which could simultaneously withstand high temperature molten oxide corrosion and oxygen evolution.^298^ Reviews on the electrolysis of iron were done recently by several authors.^298,299^ A thorough review on molten oxide electrolysis was published previously.^300^

The pursuit of an alternative aluminum electrolysis route is justified by the long-lasting difficulties to use an inert anode or bio-anode in the Hall–Héroult process. The capital expenditures and operational expenditures of pure AlCl$_3$ electrolysis were discussed recently^301^; it was reported that part of the production cost shifts from the electrolysis to the production of AlCl$_3$ while making the overall process economical. A green process should not use carbon during the chlorination of alumina to make AlCl$_3$. If carbo-chlorination is used, bio-carbon should be relatively easy to implement as, contrarily to its application as bio-anode or coke for the BF, there is no requirement on its mechanical properties for carbochlorination. Other alternative ways to produce aluminum were reviewed.^141,302–304^

There were some proof of concept experiments using direct electrochemical reduction (DER) for Fe^305^ and Al.^306^ DER is a relatively new technique which was first developed and commercialized for Ti production; a recent review on this subject was published.^307^

### Valorization of residual energy in pyrometallurgical processes

Any process involving elevated temperatures will lose some energy to its surrounding. This residual energy could potentially be recovered using different strategies which will be presented in this section. It is to be noted that the quality of the residual heat source is directly related to its temperature. In pyrometallurgical applications, output streams are often well above 650 °C and are called high-grade waste energy sources.^308^ They can be used to preheat input streams as it is the case in many ironmaking operations.^239^ In other high temperature pyrometallurgical operations (such as lead smelting and ferro-silicon production), the waste energy contained in off-gas streams is often used to generate steam used for domestic/district heating. This steam could even be used to produce electrical work via conventional Rankine cycle,^309^ which appears not to be the most energy efficient approach along with having high infrastructure costs.

Another aspect to consider in pyrometallurgy is that off-gases may still contain some chemical internal energy to be released upon post-combustion operations. As an example, blast furnace off-gases contain about 20% (volume) of CO(g) and 4% of H$_2$(g).^239^ These combustible gaseous species can be burnt in special furnaces used to preheat the air injected in the blast furnace. Flue gases may also be at sufficiently high pressure to power top pressure recovery turbines as reported by Chen et al.^310^ These authors also identified all other energy valorization opportunities that exist in the iron and steelmaking industry via the blast furnace routes; these include coking, sintering, ironmaking in BF, steelmaking in BOF and steel rolling. Even though the primary production of aluminum involves substantially lower operating temperatures than for steel production, there are also several opportunities to recover waste heat via heat exchangers, direct electrical conversion devices and steam generation as highlighted by Brough and Jouhara.^36^

#### Heat exchange technologies

One simple way to recover residual energy is to transfer it to low-temperature streams to be injected in high-temperature pyrometallurgical reactors that need to be externally heated under normal operation conditions. This energy can be transferred either directly via heat exchangers (with or without physical separation) or indirectly through the heating of inert solid materials (such as high thermal capacity refractory materials which can then transfer the energy to low-temperature gaseous streams). The heat exchanging technologies that are targeted for waste heat recovery are: air preheaters, plate heat exchangers and heat pipe systems.^311,312^ Off-gases coming from pyrometallurgical units typically consist of a mixture of inert and reactive gaseous species (which may arise from oxidizing, sulfidizing, carburizing, and chlorine-contaminated conditions^313^) along with dust particles of various natures (i.e. metals, oxides and sulfides among others). Fouling, slagging and corrosion are some common problems that need to be addressed when trying to use heat exchangers involving such chemically aggressive off-gases.^36,314^ An adequate material selection can reduce corrosion: stainless steels, nickel superalloys and refractory metals/alloys (Mo, Nb, Ta, W) are all candidate materials for moderate to high temperature conditions.^315^ These are expensive materials, which may limit the integration of heat recovery units. Maintenance and part replacement are other factors that may limit their use in pyrometallurgical operations.

#### Power cycles: Rankine and Kalina

Another approach to recover waste energy is via the generation of steam/supercritical fluids to power thermodynamic cycles such as Rankine and Kalina in order to produce electricity.^310^ These cycles have limited overall efficiencies as they involve a non-ideal heat transfer to generate steam as well as the sequential energetic conversion of the steam enthalpy into mechanical work and ultimately into electrical work. Conventional coal-fired steam power plants (classical Rankine cycle) have efficiencies between 42% and 46% according to Liu et al.^316^ These authors also showed how the efficiency of these power plants can be increased by using supercritical CO$_2$ instead of steam (i.e. the Brayton cycle). In pyrometallurgical applications, the heat source is not a flame but a much lower temperature heat source (such as off-gases). As a result, the overall energetic efficiency is typically below 30%.^309^ In many pyrometallurgical applications where the off/flue gas is below 400 °C, the organic Rankine cycle (which uses working fluids such as hydrofluoroolefins instead of water) is a possible option to circumvent the problem associated with the low temperature of the heat source.^317^ For even lower heat source temperatures (i.e. between 150 and 200 °C), the Kalina cycle operating with an ammonia-water working fluid may be an alternative option to recover energy in the form of electricity. Salemi et al.^318^ performed a technoeconomical analysis of the integration of a Kalina cycle into the direct reduction iron Midrex process. They considered a DRI exhaust gas temperature of 429.4 K in their study. They concluded that the integration of such a heat recovery technology could lead to substantial residual energy valorization. The high capital cost is the main obstacle for establishing such plants in the future.

#### Thermoelectric systems

A thermoelectric system is a device converting thermal energy into electrical work via the Seebeck effect. The temperature gradient required to induce this electron flow inside the thermocouple assembly (composed of p and n-type thermoelements) is created by exposing the thermoelectric module to a heat source (ex.: flue gas) on one side and a heat sink (ex.: water-cooled stream) on the other side.^319^ The energetic conversion of such a system is low (i.e. around 5%), which is a major drawback.^320^ This low efficiency is compensated by the simplicity of the technology which does not require moving parts, and is not noisy as well as environmentally safe.^321^ As highlighted by Araiz et al.,^319^ there are few to none large-scale deployments of this technology in the industry. Also, the compilation of Ochieng et al.^321^ shows that the maximum power output for a single unit is around 100 W for current technologies mostly implemented in automotive engines. Pyrometallurgical applications of thermoelectric systems include the industrial testing by JFE of a 10-kW class thermoelectric power generation system for its continuous casting line with KELK Ltd^322^ along with the numerical exploration of the heat recovery of other processes, including silicon casting^323^ and hot steel casting^324^ processes.

## Environmental impact mitigation

The mitigation of the environmental impacts of the pyrometallurgical industry is not the work of single actors, but rather a global effort of the entire industry. The aim is to act locally on each individual process and unit operation while thinking globally about their impacts. Taking actions that ultimately shift GHG emissions from one country to another will be of no use in the fight against climate change as the Earth’s atmosphere intermixes globally.^325^ This section presents different approaches to mitigate the environmental impact of pyrometallurgical processes.

### The 3RV principle

The 3RV principle (i.e. Reduce, Reuse, Recycle and Valorize) is a fundamental approach in environmental impact mitigation. This principle is a well-known prioritization tool that is widely used for maximizing the positiveness of actions in waste management.^326^ It could equally apply to the pyrometallurgical industry (or any heavy process). The basics of 3RV are as follows:

*Reduce* This is a no-brainer but also the *Holy Grail* when it comes to environmental impact mitigation strategies. It is valid throughout the producer-to-consumer chain (i.e. from the single citizen to the largest industrial steel producer). Any potential reduction in the consumption of raw materials and energy should be assessed during the development of new technologies or retrofit projects. For instance, the reduction (or substitution) of a harmful chemical such as CaF$_2$ used in the steel industry as a mould flux^327^ is an option that could reduce negative environmental impact and health hazards. The electrification potential of the pyrometallurgical industry in distinct regions of the world such as in Quebec (Canada) is exceptional thanks to the low-carbon footprint electricity obtained from hydropower.^328^ It has already benefited the primary aluminum and steel industry for decades now and could provide electrification opportunities to other processes including:

- Water electrolysis for H$_2$ production
- Electric-based heating systems to replace fossil fuel burners in remelting furnaces and rotary kilns
- Steam methane reforming for H$_2$ production via electric-based processes such as microwave plasma torches^329^
- Plastic recycling pyrolysis using microwave heating^330^

*Reuse* It generally refers to the reuse of any material or energy stream as is. This is a more challenging aspect with regards to the process industry because the streams are constantly transformed, therefore making it less applicable in this context.

*Recycle* This is a mature subject in the pyrometallurgical industry as metals and alloys can be infinitely remelted or re-integrated in primary metal processes (assuming that impurities can be removed or diluted). “Recycled materials as alternative feed to ores” section presented many specific examples of recycled streams already integrated into pyrometallurgical processes.

*Valorize* Both material and energy streams can be valorized. Many residual energy valorization strategies in pyrometallurgy are already implemented because of the high temperature involved, which results in high quality heat source streams (see “Valorization of residual energy in pyrometallurgical processes” section). Moreover, useful tools such as the pinch analysis^331^ enable the assessment of potential unused energy streams with sufficient quality to benefit the process. The pinch analysis technique can also be modified and used to valorize water and hydrogen usage. In recent years, tremendous efforts have also been made to reduce CO$_2$ emissions. In fact, CO$_2$ capture and valorization (i.e. chemical looping) are now being seriously considered as a potentially viable option for many industries.

### CO$_2$ capture, utilization and storage

As explained throughout this review work, carbonaceous materials represent a dominant part of the reactants/fuels used in the primary and secondary metal production processes. Therefore, getting completely rid of coke, methane and other carbon-based materials is at this time virtually impossible, especially if the industry needs to sustain the actual production intensity for most metals. One direct consequence of using carbon-based materials in pyrometallurgy is the massive production of CO$_2$. As a vivid example, this industry alone generated almost 30$\%$ of the CO$_2$ emissions of the province of Quebec (Canada) in 2018.^332^ These emissions are generally separated into five distinct sources which are (from low to high CO$_2$ concentrations): (1) ambient air, (2) post-combustion, (3) pre-combustion, (4) oxy-fuel and (5) chemical looping.^333^ “LCA analysis in pyrometallurgy” section demonstrated how the primary production of aluminum via consumable carbon anode electrolysis generates large quantities of CO$_2$ per kg of produced metal. BOF and EAF are also emitting significant amounts of CO$_2$ per kg of steel produced.

In this context, the elaboration of efficient strategies to capture, store and even use CO$_2$ emissions would represent a breakthrough for the industry. This field is now commonly referred to as either (1) carbon capture and storage (CCS), (2) carbon capture and utilization (CCU), or (3) carbon capture, utilization and storage (CCUS). CO$_2$ capture is not a new concept: this standard principle is used to separate CO$_2$ from natural gas stream in oil and gas industries as reported by Pantoon et al.^334^ The injection of the captured CO$_2$ in oil fields (a process called enhanced oil recovery) is one valorization route.^335^ The sequestration of CO$_2$ via geological injection to decrease its atmospheric concentration is a possible approach which is not considered a safe option for long term sequestration because of the high risks of massive leaks.^336^ Because of this, scientists are also exploring the possibility to perform mineral^337^ and even slag^338^ carbonation to sequester CO$_2$ in a safe and stable fashion.

Vast efforts to decrease GHG emissions in the pyrometallurgical industry brought a lot of attention to the development of CO$_2$ capture technologies. Those can be classified as follows: chemical absorption, physical separation, oxy-fuel separation, membrane separation, calcium looping, chemical looping, direct separation and supercritical CO$_2$ power cycles.^339^ Many reviews are available in the literature to track the rapid progress in this field.^340–345^ In pyrometallurgical processes, chemical absorption and physical separation are the most suitable strategies to be integrated into existing operations. CCUS would be applied to emissions from post-combustion and pre-combustion processes.

Chemical absorption capture involves a chemical reaction between a solvent and CO$_2$. The absorbed CO$_2$ is then released by increasing the solution temperature in a stripping column. Known examples of this technology are the amine-based scrubbing process (ex.: CANSOLV Technology by Shell^346^) and the enzymatic scrubbing process^347^ (ex.: CO$_2$ SOLUTIONS by SAIPEM). CO_2_ amine absorption is energy intensive due to the high temperature required to regenerate the absorbent.^345^ Commonly used absorbents are mono-ethanolamine (MEA) which requires 3.6–4.0 MJ/kg^348^ as well as diethanolamine (DEA) and N-methyldiethanolamine (MDEA).^340^ It is to be noted that amines are considered as pollutants, which explains why new absorbents based on potassium are being developed. Additives such as PZ-diamine can increase absorption kinetics and thus reduce energy consumption.^340^

Physical separation includes adsorption, absorption, cryogenic separation/dehydration and compression. During physical adsorption, CO$_2$ is adsorbed on a solid medium like activated carbon, alumina, metallic oxides or zeolite. CO$_2$ is then released by increasing temperature or pressure, respectively called temperature and pressure swing adsorption. For physical absorption, like for chemical absorption one makes use of a solvent, but no chemical reaction is involved. Temperature of the solution must also be increased to release the absorbed CO$_2$. Known solvents for physical absorption are Selexol^349^ (Honeywell UOP) and Rectisol^350^ (Linde AG and Air Liquide). Utilization of Metal-Organic Framework (MOFS) is a promising and greener solution to capture CO$_2$. The thermodynamic analysis of Maréchal shows that the energy of a truck is sufficient to have a green capture of CO$_2$, while a pyrometallurgical process has even more.^348^

Although extensive research activities are in progress in this field, the positive impact of existing CCUS technologies is still modest. In 2020, there were a little less than 30 commercial CCUS projects totalling a carbon capture capacity of approximately 45 Mt of CO_2_ annually (data of 2021).^351^ The capture capacity has tripled since 2010, but is far from the 2050 net zero scenario level of 1150 Mt CO_2_ captured per year. This includes the Boundary Dam Carbon Capture Project from SaskPower in Saskatchewan (Canada)^352^ and the Sleipner offshore gas facility in Norway.^353^ This latter project, with its capture capacity of 0.9 Mt/year, is the first large-scale CCS project which has now stored more than 20 Mt of CO_2_ in a deep saline formation since 1996. Finally, the Petra Nova facilities located in Thompsons, Texas led by Mitsubishi Heavy Industries America is another example of recent CO_2_ capture initiatives. It is one of the largest CCS plant in the world owing to its capacity of up to 1.4 Mt of CO_2_ per year with a price of 60 USD/tonne of CO_2_.

### Pre-treatment and gas scrubbing technologies

Pyrometallurgical processes involve the release of gaseous species and solid particles in the form of dust and ashes (e.g. SO_2_, NO_2_, CO_2_, CO, Hg, As-, Sb-, Se-, Te-oxides).^354^ Therefore, most pyrometallurgical plants must include a gas treatment facility and periodic measurement of dust, metals, SO$_x$, NO$_x$, and CO is required.^355^ Gas scrubbing also decreases fouling, corrosion and erosion problems of downstream components such as heat recovery units and CCU equipment. More specifically, pre-treatment of flue gases is critical for further CO_2_ utilization as some components present in off-gases may act as a poison for catalysts. For example, concentration of about 5ppm of sulphur leads to poisoning of nickel catalysts at 800 °C while only 0.01 ppm are needed to induce the same problems at 500 °C.^356^

Entrained solid particles such as metals, dust, ashes and solidified slag should be removed first as they are detrimental to the next treatment operations. Bag houses are easy solutions to remove these solid particles but, because of the high temperature generally involved in pyrometallurgical processes, other technologies such as cyclones, ceramic/metallic filters, or electrostatic precipitators are preferred.^357^ For gaseous species, different technologies exist to mitigate their release to the atmosphere. An important gaseous species to scrub in pyrometallurgy is SO$_2$. It is produced in large quantities from the oxidation of sulphide minerals and in smaller quantities from the presence of sulphur in fossil fuels. For flue gases with high SO$_2$ concentration, hydrodesulfurization (HDS) is preferred.^358^ The captured SO$_2$ can then be converted to sulphuric acid or elemental sulphur and/or reacted with lime to form gypsum.^354^ For flue gases with low SO$_2$ content, sulphur guard bed (adsorption) or chemical scrubbing (absorption) are viable options. With sulphur guard bed, the typical sulphur content at the end of the treatment is $<\sim$1 ppmv and typical catalysts would be ZnO or Cu–Zn–O.^359–361^ Catalyst companies usually have sulphur removal adsorbents in their catalogue. For chemical scrubbing, the typical sulphur content in the treated gas can be anywhere from $<\sim$1 to 20 ppm, depending on treatment conditions. Amine and caustic soda are usually the preferred solvent for this type of process. Commercial examples of sulphur treatment via chemical scrubbing are the Aminex and Thiolex process by Merichem, the Selexol process by Honeywell UOP and the Cansolv SO$_2$ scrubbing system by Shell.

NO$_x$ gases (NO and NO$_2$) are also generated by the pyrometallurgical industry mostly as the result of uncontrolled combustion operations. NO$_x$ emissions are easily spotted because of the yellow-brown gaseous fume resulting from their presence. They can be treated with different techniques: selective catalytic reduction, wet scrubbing, absorption into water and adsorption onto carbon.^362^ Selective catalytic reduction involves the introduction of NH$_3$ into the gas to break down NO$_x$ into N$_2$ and H$_2$O.^357^ Since catalysts used in this process are often poisoned by SO$_2$, care must be taken. As for wet scrubbing, compared to other acid gases, NO$_x$ scrubbers are more complicated to operate.^363^ They need longer residence time and greater packing height. They operate at high pH ($>\sim$12.5) and are not suitable for combustion exhaust gases. Also, when high CO$_2$ concentration is present, there is a competition for the solvent with NO$_x$, leading to high consumable consumption, thereby increasing the overall OPEX of the operation. However, commercial applications for NO$_x$ removal exist (see for instance Tri-Mer Corporation and MACH Engineering to name just a few). Other contaminants like mercury can also be present in the off-gases of pyrometallurgical operations, especially in processes involving battery recycling.^364^ Hg can be recovered using powdered activated carbon injection followed by a capture system like an electrostatic precipitator or a bag house.^357^ An adsorption step using iron-based adsorbents (Fe$_2$O$_3$) can also be used for Hg removal, operating at 60–100 °C.^365,366^

## Conclusions

This review showed the many ways that the pyrometallurgical industry can reinvent itself in the future to lower its impact on the environment while maintaining a high productivity. These major impacts of the industry are clearly identified in our work based on various LCAs found in the literature. Greener reactants such as bio-fuel, bio-char, hydrogen and ammonia as well as recycled feeds are already available and can potentially lower CO$_2$ emissions as a result of the replacement of carbon-based reactants and of the lowering of the energy requirement associated with recycling. Renewable energies can be valorized in pyrometallurgy mostly as a result of the electrification of different unit operations. The integration of new valorization strategies of residual energy in these processes could also positively contribute to make this industry greener. In fact, many strategies are already in place to do so (ex.: waste heat boilers which are integrated in many pyrometallurgical processes) because of the heat quality of many residual energy sources/streams. When traditional processes cannot be modified, mitigation strategies such as CCUS, gas scrubbing and industrial symbiosis need to be established. Even though there is no miracle solution to prevent the impact of the pyrometallurgical industry, the following simple guidelines could also greatly help:

- Know your process to improve your process. Lack of data and fundamental understanding of a process is a critical aspect slowing its improvement and optimization. Many industries see digital twins, data mining and machine learning as the next generation of tools to be implemented to improve energy efficiencies which typically correlate with lower CO$_2$ emissions.
- Design processes and products for an easier recycling of end-of-life products. Sorting is key to maximize the recovery of valuable metals in recycling operations. We showed here the significant effect of contaminants (such as copper in iron and aluminum scrap) and the role that scrap recovery can play in mitigating its effects.
- Participate to the elaboration of 2050 roadmap. New technologies take time to develop and projection in the future should be available to take action.
- Perform LCA of different scenarios to quantify their respective impacts and help in the decision process.

Mindset of politicians and decision makers will also have to be changed in the future if progress is to be made in this field. At the moment, many companies prefer to pay carbon taxes instead of trying to capture and sequester CO$_2$ (ex.: penalty of about 40USD per ton^367^ vs 40–100USD of capture cost with CCUS.^368^) Another solution for industries is to buy carbon allowances. These certificates to emit a ton of CO$_2$ are priced as of April 2022 at about 86USD (Europe market),^369^ which is still not a price high enough to drive the production paradigm shifts in pyrometallurgy. Future work will explore other options linked to the imposition of fair price of metal and energy based on the CO$_2$ emissions of their respective production processes to ease this transition. Finally, it has to be remembered that even though we regrouped here many common strategies, each industry (i.e. non ferrous, ferrous and aluminum) has a distinct roadmap to lower their environmental footprint. For example, reducing mining and comminution (crushing, grinding, etc) energy are priorities for the sulfide-based Cu and Ni industry, while priorities are on reduction technologies for iron and aluminum primary production.

## Data Availability

All data generated or analysed during this study are included in this published article.
